# Experimental Evaluation of a 3-D CZT Imaging Spectrometer for Potential Use in Compton-Enhanced PET Imaging

**DOI:** 10.1109/trpms.2022.3200010

**Published:** 2022-08-18

**Authors:** Yifei Jin, Michael Streicher, Hao Yang, Steven Brown, Zhong He, Ling-Jian Meng

**Affiliations:** Department of Nuclear, Plasma and Radiological Engineering, University of Illinois at Urbana–Champaign, Urbana, IL 61801 USA; H3D, Inc., Ann Arbor, MI 48108 USA; H3D, Inc., Ann Arbor, MI 48108 USA; H3D, Inc., Ann Arbor, MI 48108 USA; H3D, Inc., Ann Arbor, MI 48108 USA; Department of Nuclear, Plasma and Radiological Engineering, Department of Bioengineering, Beckman Institute for Advanced Science and Technology, University of Illinois at Urbana–Champaign, Urbana, IL 61801 USA

**Keywords:** Compton scattering, cadmium zinc telluride (CZT) detector, positron emission tomography (PET)

## Abstract

We constructed a prototype positron emission tomography (PET) system and experimentally evaluated large-volume 3-D cadmium zinc telluride (CZT) detectors for potential use in Compton-enhanced PET imaging. The CZT spectrometer offers sub-0.5-mm spatial resolution, an ultrahigh energy resolution (~1% @ 511 keV), and the capability of detecting multiple gamma-ray interactions that simultaneously occurred. The system consists of four CZT detector panels with a detection area of around 4.4 cm × 4.4 cm. The distance between the front surfaces of the two opposite CZT detector panels is ~80 mm. This system allows us to detect coincident annihilation photons and Compton interactions inside the detectors and then, exploit Compton kinematics to predict the first Compton interaction site and reject chance coincidences. We have developed a numerical integration technique to model the near-field Compton response that incorporates Doppler broadening, detector’s finite resolutions, and the distance between the first and second interactions. This method was used to effectively reject random and scattered coincidence events. In the preliminary imaging studies, we have used point sources, line sources, a custom-designed resolution phantom, and a commercial image quality (IQ) phantom to demonstrate an imaging resolution of approximately 0.75 mm in PET images, and Compton-based enhancement.

## I. Introduction

CONVENTIONAL positron emission tomography (PET) systems utilize scintillation detectors due to the relatively low cost, high detection efficiency, and excellent time resolution. However, the achievable spatial resolution of the image is limited by the intrinsic spatial resolution of the scintillation detectors, while the noise properties are significantly influenced by the energy resolution [1], [2]. Cadmium zinc telluride (CZT) is a promising room-temperature semiconductor detector material for gamma-ray detection, X-ray imaging, single-photon emission computed tomography (SPECT), and PET imaging. Large volume CZT detectors can offer an intrinsic spatial resolution of ~0.5-mm full width at half maximum (FWHM) in all three dimensions and excellent energy resolution of 5.4 keV FWHM at 511 keV [3]. Furthermore, its large volume provides the capability of detecting multiple interactions induced by a single incident gamma ray with sub-0.5-mm resolution and an ultrahigh energy resolution (3 keV @ 200 keV, 4.5 keV @450 keV) for each detected interaction, and therefore, allows the use of Compton kinematics to reject chance coincidence events. In addition, the recent research in CZT material, electronics, and data processing techniques have allowed CZT sensors to reach up to 1.5 cm in thickness in one single layer for improving sensitivity [3], which makes CZT detectors of interest for ultrahigh-resolution small-animal PET imaging.

There have been many experimental and simulation studies on clinical and small-animal PET imaging systems based on CZT/CdTe detectors. Zhang *et al*. [4] developed a prototype small-animal PET scanner based on two 3-D position-sensitive CZT detectors and experimentally achieved a spatial resolution of ~0.7-mm FWHM with a point source. Similarly, Ishii *et al*. [5] reported their achievement of ~0.8-mm FWHM resolution with a prototype CdTe-based small-animal PET scanner. Vaska *et al*. [6], [7] demonstrated sub-0.8-mm FWHM spatial resolution with a CZT-based PET scanner by simulation. Morimoto *et al*. [8] were the first to build a PET scanner dedicated to the human brain with CdTe semiconductor detectors and measured its spatial resolution of ~2-mm FWHM at the center of the field-of-view (FOV). The voxel imaging PET (VIP) system is another brain-dedicated system currently under construction, where each detector module consists of 4000 voxel channels. Preliminary simulation studies have been evaluated in [9], and [10]. Mitchell *et al*. [11] evaluated the energy resolution, intrinsic spatial resolution, time resolution, and sensitivity of a prototype PET scanner using strip CdTe detectors by simulation. Abbaszadeh’s group has also been developing a CZT-based head and neck dedicated PET system [12], [13]. The Stanford University group has developed a cross-strip CZT-based preclinical PET system and evaluated its performance which achieves an intrinsic spatial resolution of 0.76-mm FWHM [14], [15], [16], [17], [18]. Kim *et al*. [19] have developed a prototype depth-of-interaction (DOI) CZT-based small-animal PET system and a uniform subsampling DOI strategy. They achieved an ultrahigh spatial resolution of sub-0.3-mm FWHM with a point-source. Our previous work demonstrated the potential for sub-0.5-mm resolution with a CdTe-based PET prototype using a synthetic resolution point-source [20]. Finally, there also have been significant efforts in ultrahigh-resolution virtual-pinhole PET (VP-PET) using CZT detectors as inserts [21], [22], [23], [24]. In Table I, we summarize the characterization of detectors used in recent experimental research on CZT/CdTe-based PET systems.

Compared to heavy scintillation detectors, such as LSO or BGO, CZT has a relatively low density (5.9 g/cm^3^) and effective Z (~63). During PET data acquisition, there will be a large fraction of detected interactions involving Compton scattering, leading to multiple interaction sites. The previous simulation works by Xu *et al*. [25], Kolstein and Chmeissani [26], Peng *et al*. [27], and Yang *et al*. [28] have exploited Compton kinematics to recover events and to provide extra imaging information from single gamma-ray Compton interactions. The Stanford group also presented simulation studies on positioning photon multiple-interaction events, recovering interaction sequence, and rejecting random coincidence based on the CZT-PET system [16], [29], [30], [31]. Yoshida *et al*. [32] have demonstrated the so-called whole gamma imaging (WGI) that combined both Compton imaging of single photons and regular PET images. Several approaches for modeling the Compton response were developed Xu *et al*. [25] and Tashima [33].

The main objective of this research effort is to evaluate an advanced large-volume, 3-D position-sensitive CZT gamma-ray imaging spectrometer for potential Compton-PET imaging applications. *First*, we explore a CZT imaging spectrometer that offers an excellent energy resolution (e.g., ~1% at 511 keV), a sub-0.5-mm intrinsic spatial resolution in 3-D, and could detect multiple simultaneous gamma-ray interactions and precisely determine their corresponding 3-D locations and energy depositions. These features make these 3-D CZT detectors particularly attractive for Compton-PET imaging applications. *Second*, we have developed a Monte-Carlo-based numerical integration technique to derive the near-field Compton response of the 3-D CZT detectors. *Finally*, we constructed a prototype PET system based on the 3-D CZT imaging spectrometers and experimentally evaluated the spatial resolution and energy resolution of the system, and the effect and accuracy of Compton-based random rejection.

## II. Material and Methods

### A. 3-D CZT Imaging Spectrometer

The prototype PET system is constructed using CZT imaging spectrometer modules that each has a monolithic CZT crystal of 2.2 × 2.2 × 1.0 cm^3^ in size. The CZT detector has 11×11 anode pixels of 1.9-mm pitch and a large continuous cathode [35]. This detector uses a unique design that: 1) measures the charge-drifting time inside the detector to provide the DOI information; 2) utilizes relatively large (1.9 mm) anode pixels to ensure an excellent energy resolution (e.g., 5.4 keV at 511 keV) while using the transient signals induced on adjacent anode pixels to achieve a sub-0.5-mm intrinsic spatial resolution; and 3) detects multiple simultaneous gamma-ray interactions and precisely determine their corresponding 3-D locations and energy depositions. The basic design concepts for the CZT detector are presented in [34], [35], [36], [37], and [38]. These features make these 3-D CZT detectors particularly attractive for Compton-PET imaging applications.

### B. Prototype PET System Setup

**Fig. 1.**
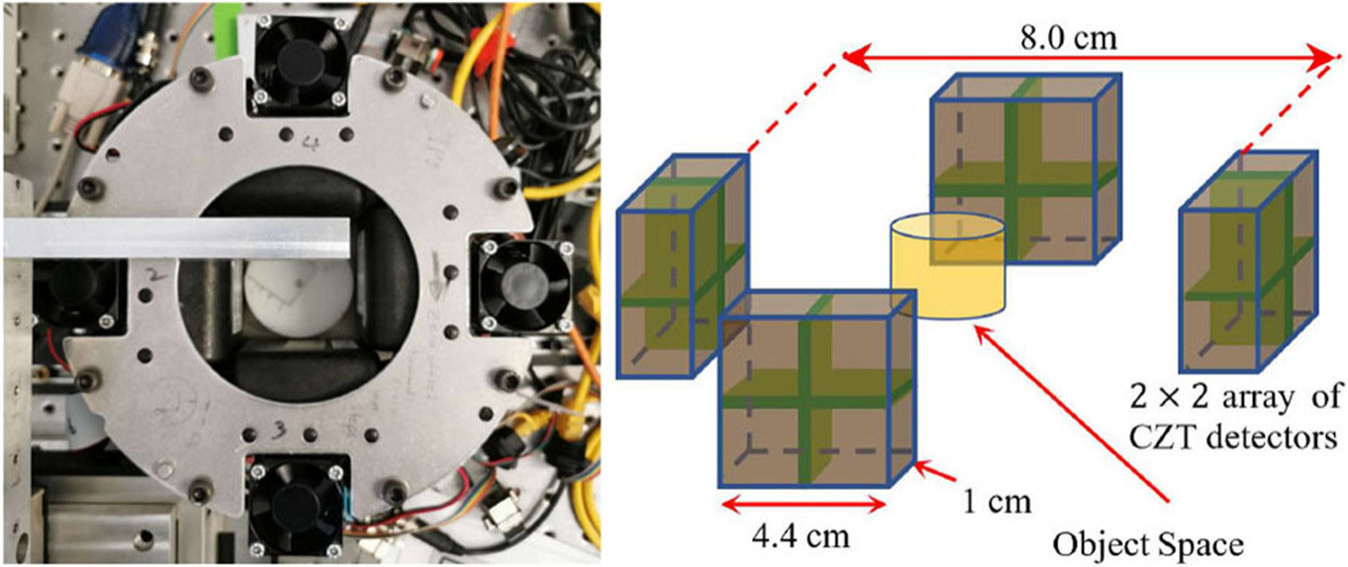
(Left) Prototype CZT-PET system and (Right) schematic and dimensions of the CZT-based detection system.

The schematic of the prototype PET setup is shown in Fig. 1. The system consists of four CZT detector panels. Each panel has four (2 × 2) CZT detector modules (detailed in Section II-A), offering a detection area of around 4.4 cm × 4.4 cm. The distance between the front surfaces of the two opposite CZT detector panels is about 80 mm. The position, energy, and timing information of each detected gamma-ray interaction are saved in a list-mode dataset. The coincidence pairs are determined in post-processing through the timestamp of the individual detected events. A high-resolution rotation stage is used to support the object accompanying with 3-D linear translation stages to adjust its position, which allows the source to be rotated within the coincidence detection system to collect projections from multiple angles while the four detector panels are stationary.

It is worth noting that the CZT detectors used in this prototype rely on the triggering from the anode (pixel) signal to derive the timing information. Given the small pixel effect resultant from the pixel geometry, the triggering signal is delayed for up to a few hundred ns from the actual interaction time, and the length of the delay depends on the DOI inside the 1 cm thick crystal. In this experimental study, we have *not* implemented the timing correction based on interaction depth. Therefore, the coincidence timing resolution of this prototype system is around 100 ns, which severely limits the count rate capability of this system. In the experimental studies reported in this article, we have limited the activity of the object to <25 *μ*Ci to avoid excessive chance coincidence.

To further improve the timing resolution of the large-volume CZT detectors, we have previously reported both theoretical and experimental results on using the cathode signal to extract the interaction timing, which could improve the timing resolution to approximately 10 ns for CZT detectors of 1 cm in thickness [39], [40].

### C. Modeling of Sensor Response

#### 1) Modeling of the CZT-Based Coincidence Detection System:

The CZT detector module was modeled with multiple (i.e., 1, 2, 4, and 10) layers across its 1 cm thickness, and 100 × 100 voxels in lateral directions, so the dimension of each detector-voxel is 209 *μ*m × 209 *μ*m × 0.1 − 1 cm in size.

**Fig. 2.**
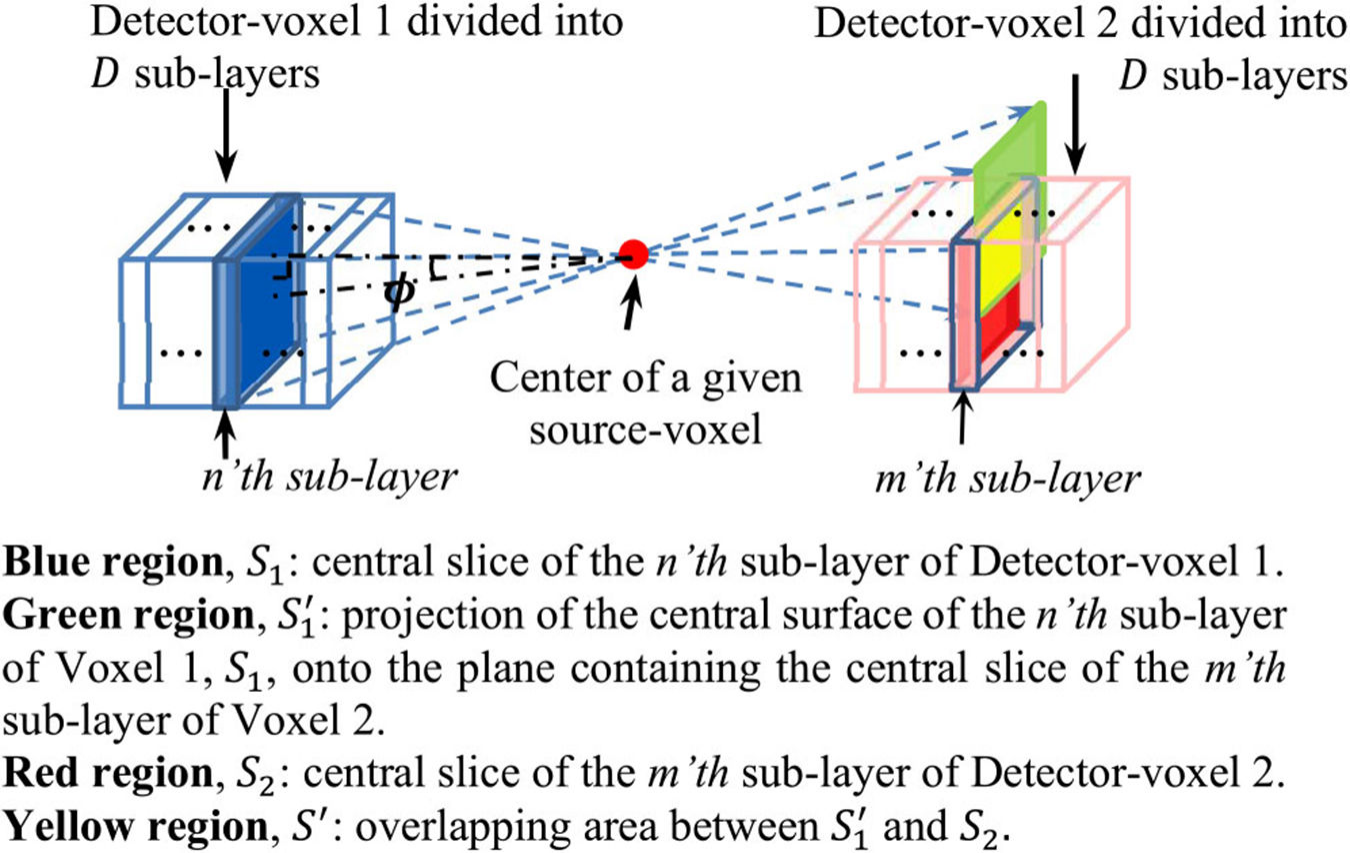
Illustration of the calculation of the probability $p(A_{j}|i)$.

Consider a coincidence event A denoted by ($I_{1}$, $I_{2}$), where $I_{1}$ and $I_{2}$ are the indices of detector-voxels belonging to two opposite detectors, in which the two gamma rays from a positron annihilation are detected (Fig. 2). These two detector-voxels are referred to as detector-voxels 1 and 2 in the following discussion. Since the thickness of the detector-voxels (along the 1 cm thickness, which will be referred to as the *Z*-direction) is large compared to its dimensions in the other two directions (referred to as the *X*- and *Y*-directions), we subdivided each voxel into *D* sublayers along the *Z*-direction as shown in Fig. 2.

The probability of the coincidence event A originated from a given source-voxel i is calculated by combining the probabilities of the event being detected by each possible pair of sublayers selected from both detector-voxels that detected the given coincidence event

(1) $$p(A\;|\;i)=p(I_{1},\;I_{2}|i)\;\;=\sum_{m}^{D}\sum_{n}^{D}p(I_{1},n|i)\cdot p(I_{2},m|i,I_{1},n)$$

where $p\;(I_{1},n\;|\;i)$ is the probability that the first annihilation gamma-ray emitted from source-voxel i is detected at the n’th sublayer of the detector-voxel 1, while $p(I_{2},m|i,I_{1},n)$ is the conditional probability that the second gamma ray from the same annihilation is detected by the m’th sublayer of detector-voxel 2.

The probability, $p(I_{1},n|i)$, was evaluated by considering the solid angle and the attenuation of the gamma ray through the detector volume, which gives

(2) $$p(I_{1},n|i)=\frac{S_{1}\cos\phi }{4\pi d^{2}}\cdot e^{-\mu l}\cdot \left(1-e^{-\mu l_{0}}\right)$$

where $S_{1}$ is the area of the central slice of the n’th sublayer of detector-voxel 1 (shown in Fig. 2), d is the distance between the centers of the given source-voxel i and detector-voxel 1, and ϕ is the angle of incidence for the gamma-ray originated from the given source bin i reaching the n’th sublayer of detector volume within detector-voxel 1 (see Fig. 2). In addition, l is the distance for the gamma ray to travel inside the CZT module before reaching the n’th sublayer within detector-voxel 1. $l_{0}$ is the path length of the same gamma ray within the sublayer, and μ is the linear attenuation coefficient of CZT.

To derive the conditional probability, $p(I_{2},m|i,I_{1},n)$, we first back-projected the central slice of the n’th sublayer within detector-voxel $r_{1}$, through the origin of the gamma ray, onto the plane within detector 2 that contains the central slice of the m’th sublayer of detector-voxel 2. We then computed the overlapping area (denoted by *S*′) between the projection and the central slice of the m’th sublayer of detector-voxel 2. Then, the conditional probability $p(I_{2},m|i,I_{1},n)$ can be derived as

(3) $$p(I_{2},m|i,I_{1},n)=\frac{S^{\prime }}{S_{2}}\cdot e^{-\mu }\cdot \left(1-e^{-\mu \;\;l_{0}^{\prime }}\right)$$

with $l^{\prime }$ and $l_{0}^{\prime }$ being defined similarly to l and $l_{0}$ for (2).

### D. Modeling of the Compton Response of the CZT Detectors

**Fig. 3.**
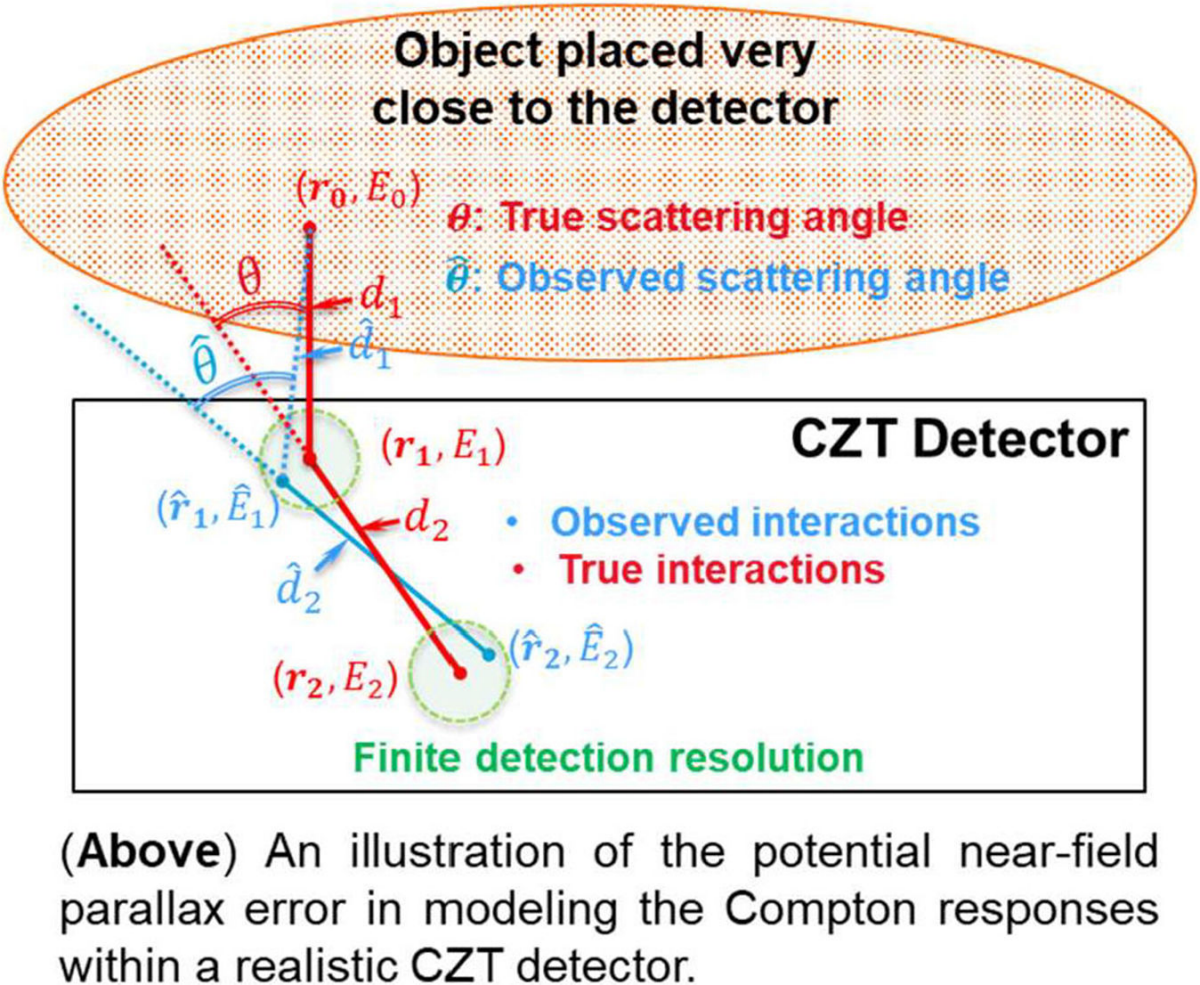
Illustration of the near-field Compton imaging problem and the potential parallax error in modeling the Compton responses within a realistic CZT detector.

In this study, we focused on the near-field Compton detection geometry, as shown in Fig. 3, where the distances $d_{1}$ and $d_{2}$, denoting the *true* distances between the source and the first interaction site and between the first and the second interaction sites, respectively, are relatively small compared to the physical dimension of the detector. In this case, the experimentally observed Compton response of the CZT detector could be markedly different from the classical Compton responses derived from the Klein–Nishina formula [41]. Yin *et al*. [22] have developed an analytical approach to quantify the angular uncertainty based on Compton kinematics and incorporated the position and energy resolution of CZT detectors. Recently, Yin *et al*. [23] modeled the angular deviation of the Compton cone as an asymmetric Gaussian function considering the energy resolution. However, Doppler broadening was neglected in their modeling.

In this work, we modeled the near-field Compton response of the CZT detector by considering both the finite detector energy and spatial resolutions, detailed gamma-ray interaction physics (i.e., Doppler broadening), as well as the distance ${\hat{d}}_{2}$ between the first and second interaction sites. The probability, p, for detecting a Compton event induced by a gamma ray from a given source-voxel could be given by

(4) $$p=p^{\prime }\cdot p_{1}\cdot p_{2}.$$

In (4), $p^{\prime }$ is the probability of the gamma-ray penetrating the media between its origin and a given detector-element, which can be readily evaluated based on the known geometry of the source and the detector. $p_{1}$ is the probability that a gamma ray from the source-voxel interacted in a detector- element through Compton scattering, *without* considering the attenuation of the gamma ray while traveling between its origin and the detector-element. $p_{2}$ is the probability of the gamma ray, known to have scattered in detector-element 1, leading to a photoelectric absorption in detector-element 2.

**Fig. 4.**
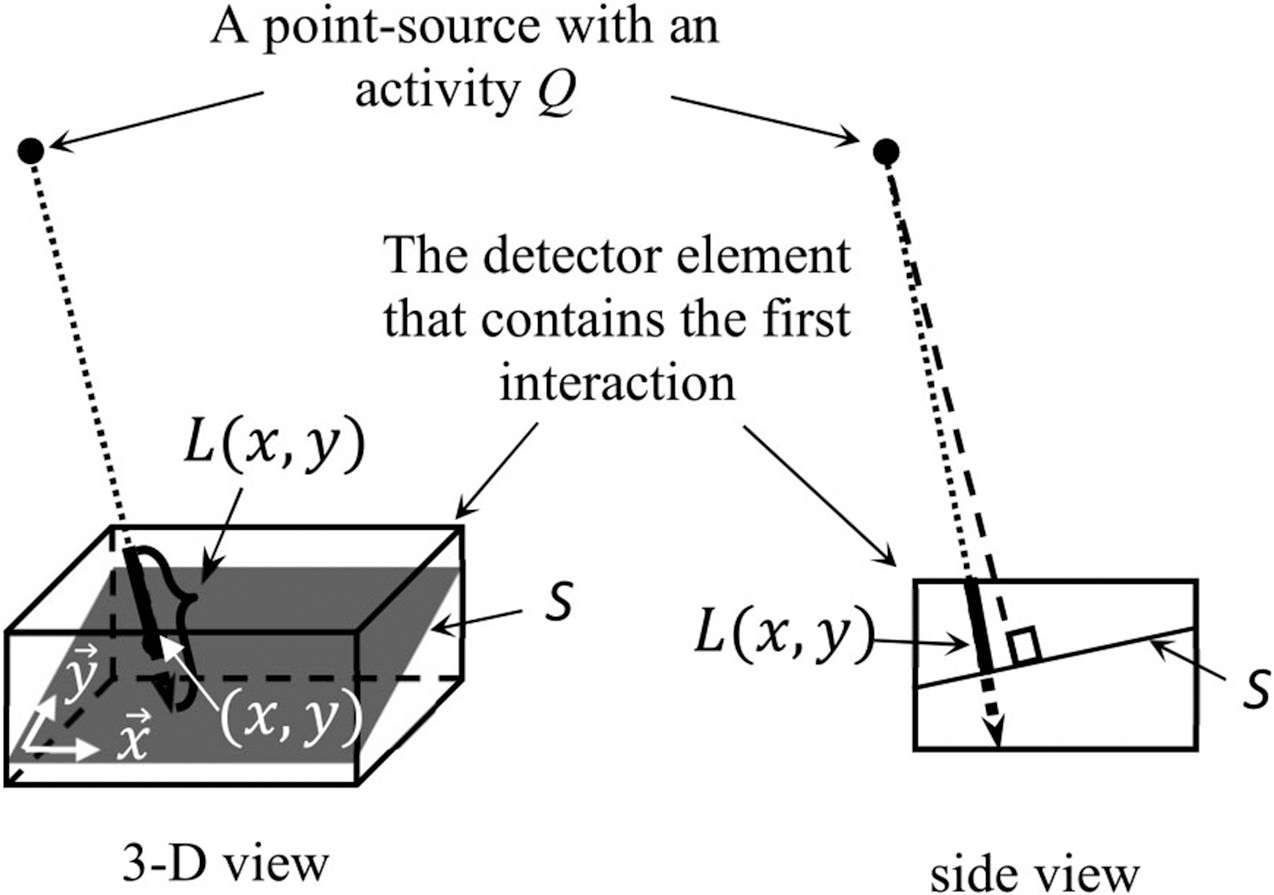
Illustration of calculating the probability that a gamma ray originated from a given source bin interacted with a detector-element through Compton scattering.

To evaluate $p_{1}$, we first considered a source-voxel with an activity $Q\;[s^{-1}]$. The cross section of a given element in the CZT detector, with respect to the source-voxel, is S [cm^2^] as shown in Fig. 4. We used {$\vec{x}\vec{y}$} to denote the bases of the plane containing the cross section area S, and L(x,y) to denote the path length of a gamma-ray beam from the source-voxel traveling inside the detector-element through the point (x,y). We assumed that the Compton scattering cross section of a CZT molecule is σ [cm^2^] and the molecular density of CZT is $n_{0}$ [cm^−3^]. Without considering the attenuation of gamma rays before reaching a given detector-element, the flux of gamma rays from the source-voxel and incident on the detector-element could be given by $\phi_{1}\;=\;(Q∕[4\pi d_{1}^{2}])[(s\cdot {\text{cm}}^{2}{)}^{-1}]$, where $d_{1}$ is the distance between the center of the source-voxel to the detector-element 1 that contains the first interaction.

In the detection geometry illustrated in Fig. 4, the number of gamma rays that would interact in detector-element 1 through single Compton scattering interactions per unit time is approximately given by

(5) $$R≅\iint_{S}\phi_{1}\;n_{0}\;\sigma \;l(x,y)dx\;dy\;\;=\frac{Q\;n_{0}\sigma }{4\pi d_{1}^{2}}\int \int_{\;S}L(x,y)dx\;dy\;[s^{-1}]$$

where S is the cross section area of detector-element 1 in respect to the source-voxel as shown in Fig. 4.

Without considering the attenuation of the gamma ray before reaching the target detector-element, the probability of a gamma-ray originated in a given source-voxel and Compton-scattered in a given detector-element is given by

(6) $$p_{1}=\frac{R}{Q}=\frac{\frac{Q\;n_{0}\sigma }{4\pi d_{1}^{2}}\iint_{}L(x,y)dx\;dy}{Q}\;\;=\frac{n_{0}\sigma }{4\pi d_{1}^{2}}\int \int_{{\;}_{S}}\;L(x,y)dx\;dy$$

where $\iint_{S}l(x,y)dx\;dy$ is essentially the volume of the detector-element V. Hence, (6) is simplified to

(7) $$p_{1}\;=\;\frac{n_{0}\sigma }{4\pi d_{1}^{2}}V.$$

To evaluate $p_{2}$, which is the probability of the gamma ray, known to have scattered in detector-element 1, leading to a photoelectric absorption in detector-element 2, we have developed a numerical integration technique that uses a large number of Compton interaction events generated with GEANT4 simulations [42] to model the Compton response of the CZT detector. In this study, we simulated a CZT detector of 4 cm × 4 cm × 1 cm in size and a pencil beam of gamma rays normally incident on the detector through the center of the 4 cm × 4 cm front surface. All the gamma rays had initial energy E_0_. In the simulation, we only considered gamma rays, each having a Compton scattering followed by a photoelectric absorption and leading to a total deposited energy equal to the initial energy of the incident gamma ray, $E_{1}+E_{2}=E_{0}$. A total of 2 billion Compton events were used to generate the Compton response function.

To account for the finite detector resolutions, we have blurred the *true* interaction position ($r_{1}$ and $r_{2}$) and energy deposition ($E_{1}$ and $E_{2}$) recorded in GEANT4 simulation to generate the *observed* interaction positions (${\hat{r}}_{1}$ and ${\hat{r}}_{2}$) and energy depositions (${\hat{E}}_{1}$ and ${\hat{E}}_{2}$), according to artificially defined resolution functions, such as 1-D Gaussian function for energy blurring and 3-D Gaussian for spatial burring. Using the *observed* interaction positions, we further computed the *observed* scattering angle $\hat{\theta }$ and the *observed* distance between the first and second interactions ${\hat{d}}_{2}$.

From these Compton events generated in GEANT4, we binned the corresponding $\left({\hat{E}}_{1},\hat{\theta },{\hat{d}}_{2}\right)$ values into a discrete 3-D matrix, F, spanned by $\left\{{\hat{E}}_{1},\hat{\theta },{\hat{d}}_{2}\right\}$ and with bin sizes of $\Delta \hat{E}=(1∕3)\;\text{keV}$, $\Delta \hat{\theta }=(1∕3{)}^{\circ }$, and $\Delta {\hat{d}}_{2}=0.25$ mm. The number of events falling into each bin of F was then divided by the total number of events in the matrix. Therefore, the (i, j, k’)th element of matrix F describes the probability that a gamma ray, known to Compton-scattered in a detector-voxel, leads to a detected Compton event with observed signatures $\left({\hat{E}}_{1},\hat{\theta },{\hat{d}}_{2}\right)$ falling into their corresponding bins

(8) $$p_{2}\left(\begin{array}{c} {\hat{E}}_{1}\in \left[i\times \Delta {\hat{E}}_{1},\;(i+1)\times \Delta {\hat{E}}_{1}\right),\\ \hat{\theta }\in [j\times \Delta \hat{\theta },\;(j+1)\times \Delta \hat{\theta }),\\ {\hat{d}}_{2}\in \left[k\times \Delta {\hat{d}}_{2},\;(k+1)\times \Delta {\hat{d}}_{2}\right) \end{array}\right)=F(i,\;j,k).$$

Substituting (7) and (8) into (4), we could evaluate the probability of a gamma-ray originated in a given source-voxel leading to an observed 2-interaction Compton event, characterized by

$$\begin{array}{c} {\hat{E}}_{1}\in \left[i\times \Delta {\hat{E}}_{1},\;(i+1)\times \Delta {\hat{E}}_{1}\right)\\ \;\;\;\;\hat{\theta }\in [j\times \Delta \hat{\theta },\;(j+1)\times \Delta \hat{\theta }),\;\text{and}\\ {\hat{d}}_{2}\in \left[k\times \Delta {\hat{d}}_{2},\;(k+1)\times \Delta {\hat{d}}_{2}\right) \end{array}$$

as

(9) $$p=p^{\prime }\cdot p_{1}\cdot p_{2}=p^{\prime }\cdot \frac{n_{0}\sigma }{4\pi d_{1}^{2}}\cdot V\cdot F(i,j,k).$$

**Fig. 5.**
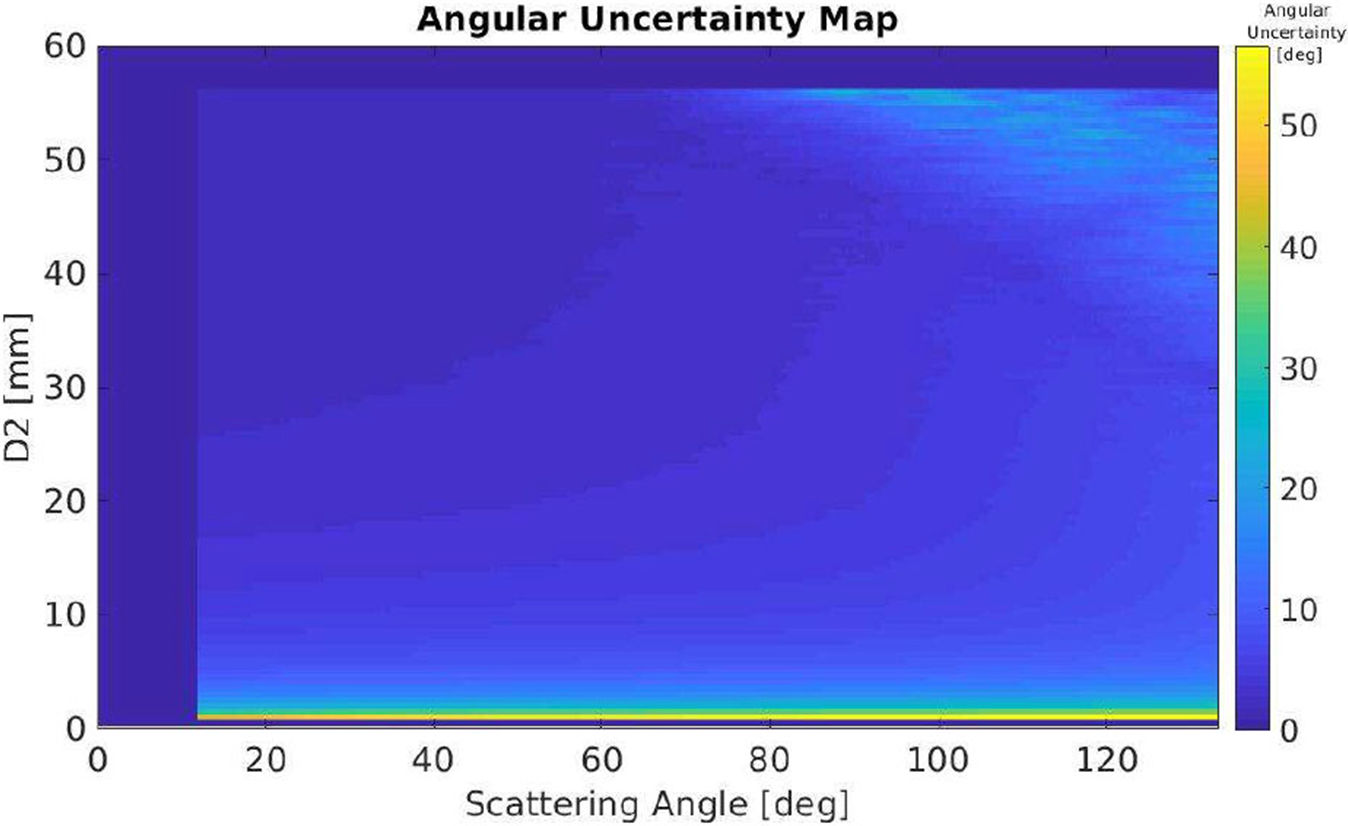
FWHM angular uncertainty map of the proposed near-field Compton model at different ${\hat{d}}_{2}$ and scattering angle.

**Fig. 6.**
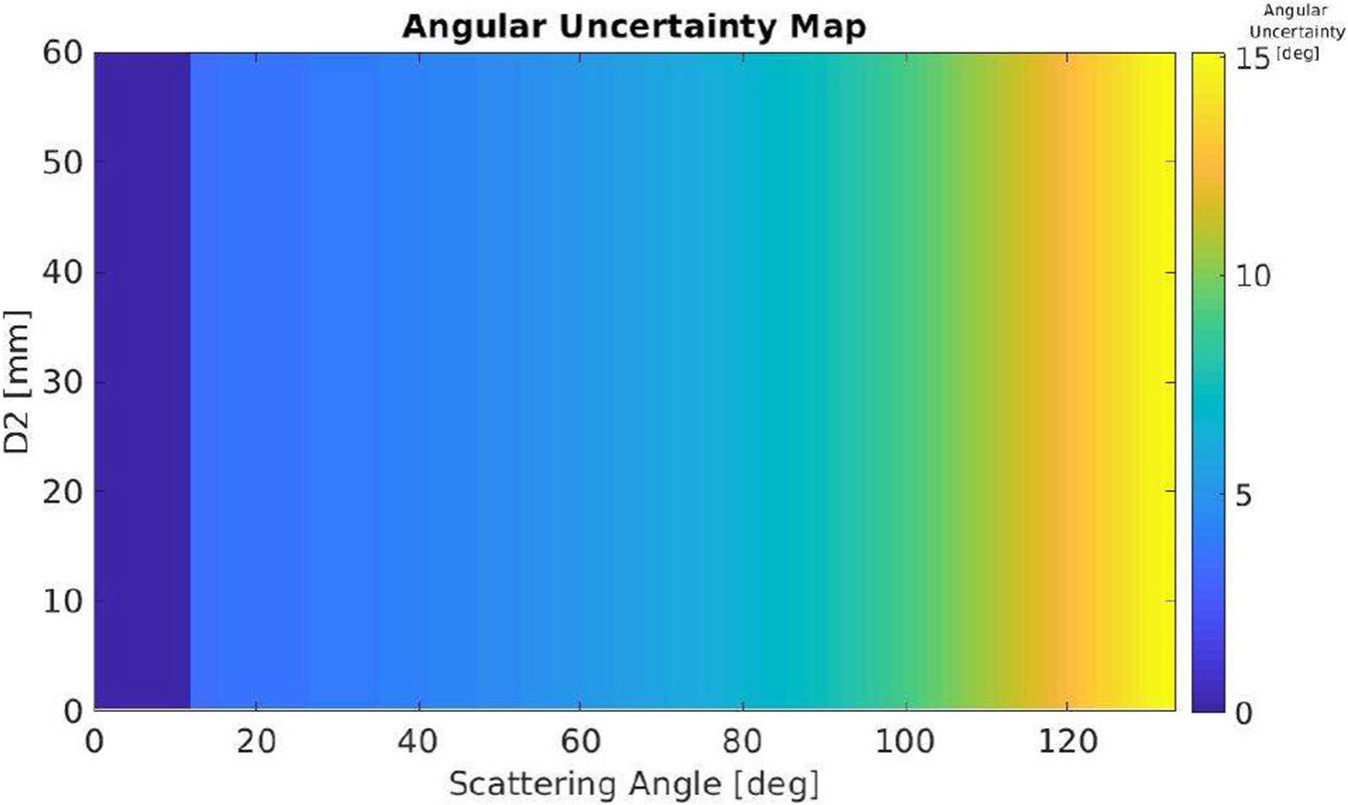
FWHM angular uncertainty map of the near-field Compton model if ${\hat{d}}_{2}$ is not considered.

An example of the influence of the Compton model that we derived in (1)-(9) is shown in Figs. 5 and 6. In this example, we compared the Compton response function of the 1 cm thickness CZT with (Fig. 5) and without (Fig. 6) considering the finite distances between the first and second interaction sites. Based on the near-field Compton response matrix F, and considering a fixed energy deposition ${\hat{E}}_{1}$, we computed the distribution of the observed scattering angle $\hat{\theta }$ and a given the distance between the two interactions ${\hat{d}}_{2}$, we have a subset group of events whose observed scattering angle will formulate a Gaussian-like distribution. We fitted the distribution by Gaussian function and derived the FWHM as the angular uncertainty at different combination of $\hat{\theta }$ and ${\hat{d}}_{2}$, as shown in Fig. 5. If ${\hat{d}}_{2}$ is not considered, in which case the dimension of ${\hat{d}}_{2}$ eliminates in the matrix F, the angular uncertainty will only change when the scattering angle changes as shown in Fig. 6. The remarkable difference demonstrates that even with Doppler broadening, ignoring ${\hat{d}}_{2}$ still has a significant influence on the accuracy in predicting the scattering angle from experimental data, in turn, leading to the degradation in the reconstruction of interaction sequence and random rejection.

### E. Using Compton Kinematics to Predict the First Interaction Site and Reject Random and Scattered Coincidences

Considering a detected coincidence event, in which at least one of the coincidence gamma rays was detected as a Compton event in its corresponding CZT detector, one may be able to use the Compton response function derived in Section II-C to determine the site of Compton interaction. This possibility has been explored in [16], [25], [26], [28], and [32] using Monte Carlo simulations, or experimental studies using non-CZT-based detectors. In this study, we experimentally evaluated the effect of using Compton kinematics for predicting the first interaction site and rejecting random coincidences using the CZT-PET prototype system.

For a 511-keV gamma ray to interact in CZT, there would be, in principle, 83% of chance that the gamma ray would undergo Compton scattering. However, within all events that we experimentally acquired with the 1-cm thickness CZT detectors, we observed only 21% out of all events having 2 or more interactions. This discrepancy is due to several physical aspects. First, each CZT detector has 11 × 11 pixels on its anode side with a large pitch of 1.9 mm × 1.9 mm. For most of the Compton interactions that have both the first and second interactions occurred within the 1.9 mm × 1.9-mm pitch, the detector cannot differentiate the two interactions, so these events were counted as single-interaction events. Second, for gamma rays that Compton scattered in the detector and then, escaped from the detector, these events will not contribute to the detected Compton events.

**Fig. 7.**
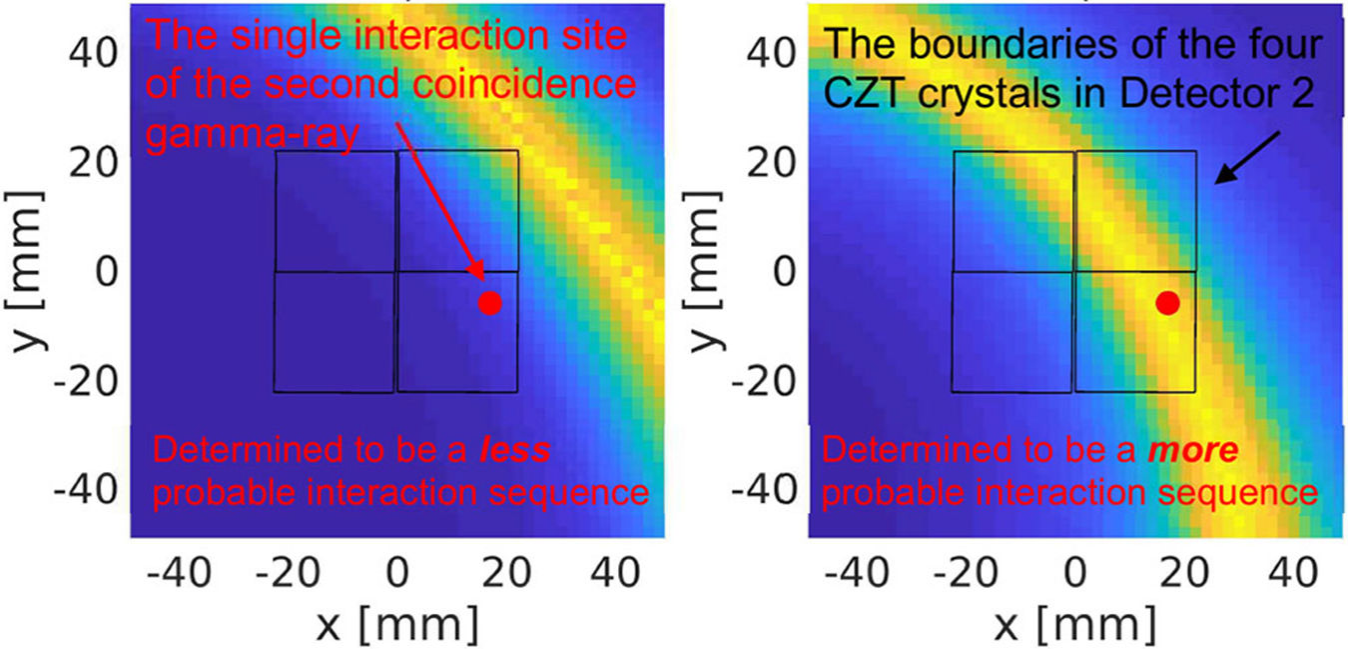
Illustration of using Compton kinematics to determine the interaction sequence (in terms of which is the first Compton interaction site among the two interactions detected on Detector 1). The colored bands are the Compton cones projected from the Compton event in Detector 1 onto Detector 2, where the second coincidence gamma-ray resulted in a single interaction as marked by the red dots.

Fig. 7 shows an analysis of an experimentally acquired coincidence event, in which the first gamma-ray interacted in Detector 1 through a Compton-scattering followed by a photoelectric absorption, and the second gamma ray is photoelectrically absorbed in Detector 2.

From the Compton interaction observed in Detector 1, we used the Compton response function described in Section II-C to evaluate the probability of the first gamma-ray reaching Detector 1 from an arbitrarily given angle. By assuming that the two annihilated gamma-rays travel at 180° back-to-back, we further projected this angular distribution into the space containing Detector 2, in which the second coincident gamma ray was detected. In Fig. 7, we plotted this angular distribution in a plane cutting cross Detector 2 and intersected the measured interaction position of the second gamma ray. The left and right panels in Fig. 7 show the angular distributions projected across Detector 2, derived by assuming one of the two possible interaction sequences that could have happened in Detector 1.

The red dots in Fig. 7 show the experimentally observed gamma-ray interaction location in Detector 2. Assuming a perfect collinearity between the annihilation photons, we concluded that the second interaction sequence in Detector 1, corresponding to the angular distribution shown in Fig. 7 (right), is more probable than the first interaction sequence shown in Fig. 7 (left).

**Fig. 8.**
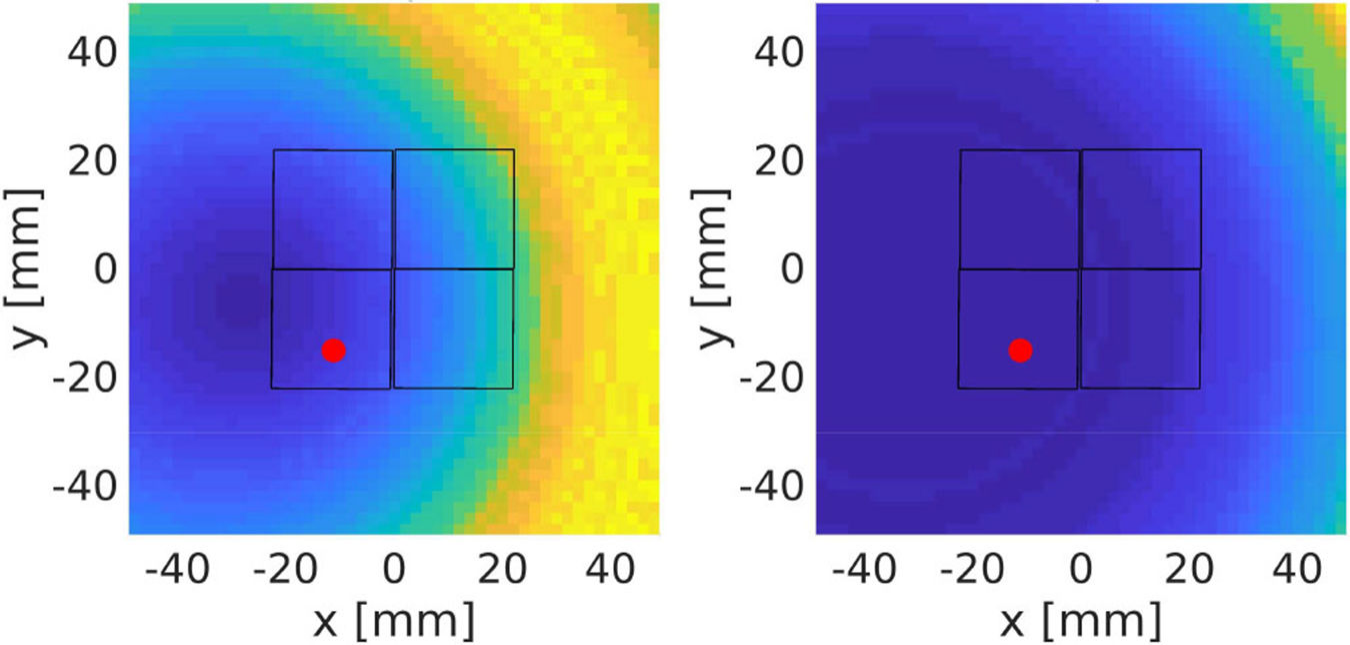
Illustration of using Compton kinematics to reject chance-coincidence events. For both possible sequences, given the Compton cones derived by the Compton event in Detector 1, the probabilities of detecting the second coincidence gamma ray at the location marked by the red dots in Detector 2 are too low. Hence, this event is rejected as chance coincidence.

In Fig. 8, we used another experimentally acquired event to illustrate the use of Compton kinematics to reject random and scattered coincidence events. In this event, Detector 1 registered a two-interaction Compton scattering event, and Detector 2 registered a single-site 511-keV full-energy interaction. From the Compton event detected in Detector 1, we derived the probability of the second gamma ray being detected by any given pixel in Detector 2. Note that we considered both of the possible interaction sequences that could happen in Detector 1.

In this example, given the first gamma ray being detected as a Compton scattering in Detector 1 with a fixed energy deposition of the first-interaction site and the distance between the two interactions, the unnormalized probability mass function of the observed scattering angle will be given by (9) by fixing i and k. For the convenience of calculation, we normalized the function by its maximum, so the probability used in the following calculation is not the actual probability but a relative value. Hence, the chance of detecting the second coincidence gamma ray at the actual detected location (marked by the red dot) is p=0.08 or 10^−4^ for the two possible interaction sequences, respectively. A threshold of 0.2 is set to reject chance coincidence events. From this derivation, we determined that this experimentally measured event is either a random coincidence or a scattered coincidence where one of the annihilation gamma rays has scattered before reaching the CZT detectors. This is a typical noise event that could be rejected with the Compton analysis.

The threshold has a significant influence on random rejection based on Compton information. A higher threshold rejects more chance coincidence as well as more true coincidence. To optimize the threshold, we used NECR as defined below for comparing different models of Compton response

(10) $$\text{NECR}=\frac{(\text{true count rate}{)}^{2}}{\text{total count rate}}$$

where the true count rate is the count rate of true coincidence events, which is unknown in the experiment.

For this specific application in Compton-based rejection, we write the NECR as

(11) $${\text{NECR}}^{\prime }=\frac{(\text{True count rate}\cdot (1-\text{false rejection rate}){)}^{2}}{\text{total count rate}\cdot (1-\text{rejection rate})}\;\;=\text{NECR}\frac{(1-\text{false rejection rate}{)}^{2}}{1-\text{rejection rate}}$$

where the rejection rate is the ratio of number of rejected events to total events, and the false rejection rate is the ratio of number of rejected true coincidence events to total true coincidence events. Obviously, the false rejection rate is still unknown experimentally, but we can estimate it by p (false rejection), the probability of incorrectly rejecting true coincidence.

Given a *true* coincidence event having a Compton scattering on one side, with the energy deposition ${\hat{E}}_{1}\in [i\times \Delta {\hat{E}}_{1},\;(i+1)\times \Delta {\hat{E}}_{1})$ of the first interaction site and the distance between the two interactions ${\hat{d}}_{2}\in [k\times \Delta {\hat{d}}_{2},\;(k+1)\times \Delta {\hat{d}}_{2})$, the probability for this event to be rejected is the area below the threshold in the probability mass function over its total area, that is

(12) p((false rejection∣E^1,d^2)=∑j1F(i,j1,k)∑jF(i,j,k)j1s.t.F(i,j1,k)<threshold⋅maxjF(i,j,k).

Note that the other terms except F(i,j,k) are canceled if we plugin (9).

The law of total probability gives that

(13) p(false rejection)=∑E^1,d^2p((false rejection∣E^1,d^2)⋅p(E^1,d^2)

where

(14) $$p\left({\hat{E}}_{1},\;{\hat{d}}_{2}\right)=\frac{\sum_{j}F(i,j,k)}{\sum_{i}\sum_{j}\sum_{k}F(i,j,k)}.$$

With regard to the accuracy of this Compton-based random and scattered coincidence rejection method, the false rejection or true acceptance rate (false rejection rate + true acceptance rate = 1), cannot be demonstrated by experiment as mentioned before, but the true rejection or false acceptance rate (false acceptance rate + true rejection rate = 1), referring to whether a chance coincidence event is rejected successfully, can be evaluated by experiment. In the experiment, we roughly know where the phantom is through reconstruction. If all the possible lines-of-response for a coincidence event determined through Compton kinematics do not pass the hot region, it would be determined as a random or scattered coincidence event. After applying Compton-based rejection to these known-to-be-random events, we can obtain the true rejection rate.

In Figs. 5 and 6, we have compared the angular uncertainty of the near-field Compton model and a conventional Compton model in which we have Doppler broadening added but ignore ${\hat{d}}_{2}$. Having the methods of estimating NECR and accuracy, we are able to compare the influence of using these two models in Compton-based random and scattering rejection.

### F. Experimental Calibration of System Geometry

In this study, we used experimentally acquired coincidence events to derive the geometrical parameters of the CZT-PET detection system. This calibration process has previously been developed and experimentally verified in [19] and [20], which has allowed us to achieve an experimental PET imaging resolution of less than 250 *μ*m with ultrahigh-resolution CdTe detectors. This method is briefly described as follows.

For each detector panel consisting of 2 × 2 CZT detector modules, we first defined a local coordinate system within a global coordinate system and used six parameters to characterize the origin and orientation of the local system within the global system. Since each detector panel comprises of four detector modules (each has a CZT crystal of 2.2 cm × 2.2 cm × 1 cm), we used six parameters to describe the position and orientation of each CZT module/crystal inside the local coordinate system, which leads to 4× six parameters to define the geometry of each detector panel, and a total of 4 × (6 + 24) parameters for the 24 CZT modules/crystals within the four detector panels. In addition, we used seven parameters to describe the orientation and position of the sample rotation stage and rotation radius, and three parameters to describe the direction of the vertical linear translation stage. This geometrical definition leads to a total of 130 parameters to define the prototype PET setup. These system parameters will be represented by a vector α.

To evaluate these geometrical parameters, we used a Na-22 point source with a 0.25–0 mm diameter mounted on the rotation stage and positioned at roughly 5-mm away from the rotation axis. We rotated the source at eight uniformly spaced angles across 360°, and then, translated the rotation stage along the rotation axis to acquire a total of 24 projections. For this calibration procedure, we used coincidence events with 511 keV full-energy deposition only. All the events were stored in a list-mode dataset that is denoted by a vector $D_{c}$.

By incorporating the detector response derived in Section II-C, and the prior knowledge that the source is a uniform sphere of 250 *μ*m in diameter, we can derive the conditional probability $p(D_{c}\;|\;\alpha )$. The most probable system parameters, $\hat{\alpha }$, can be found by constrained minimization process

(15) $$\hat{\alpha }=\text{argmin}\{-p(D_{c}\;|\;\alpha )\}$$

which is performed in MATLAB [43] using the FMINCON function.

### G. Image Reconstruction

From the measured list-mode data, we reconstructed the PET images using the list-mode ordered-subset expectation-maximization (OS-EM) algorithm [44], [45]

(16) $$f_{k}^{\left(t+1\right)}=\frac{1}{s_{k}}\sum_{j=1}^{N}\frac{p(A_{j}\;|\;k)f_{k}^{\left(t\right)}}{T\sum_{i=1}^{M}p(A_{j}\;|\;i)f_{i}^{\left(t\right)}}$$

where $f_{i}^{\left(t\right)}$ denotes the activity at source-voxel i after the $t^{\prime }\text{th}$ iteration. $A_{1},\ldots ,A_{N}$ denote the measured list-mode coincidence events. The total number of source-voxels is M. $s_{i}$ is the probability that a pair of annihilation photons emitted from source-voxel i is detected by the detection system as a coincidence event. T is the acquisition time. $p(A_{j}\;|\;k)$ is the probability of a detected coincidence event $A_{j}$ being originated from source-voxel k.

As we discussed in Section II-B, we rotated the source object at several angular steps with a fixed interval to collect projection data. They were naturally grouped as several subsets in the OSEM reconstruction.

### H. Phantom Study

**Fig. 9.**
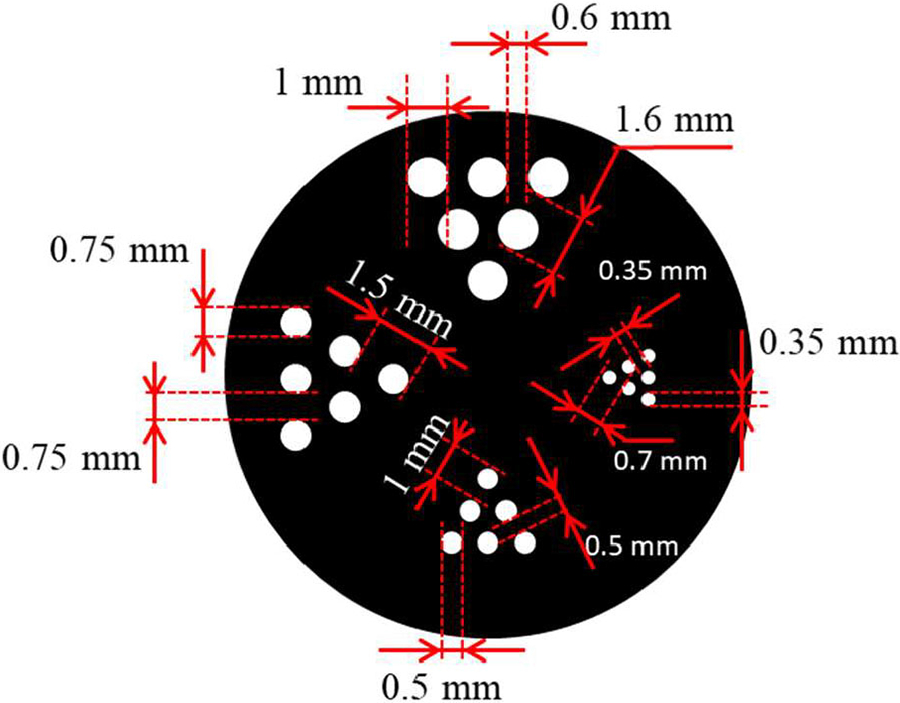
Schematic of the custom-made hot-rod resolution phantom containing four groups of hot rods of 0.35, 0.5, 0.75, and 1 mm diameter. The center-to-center distances of each two adjacent rods are 0.7, 1, 1.5, and 1.6 mm, respectively.

We carried out a series of preliminary imaging studies with: 1) a Na-22 point source of 0.25-mm diameter; 2) two line sources fabricated with two capillary tubes of 0.65-mm inner diameter filled with Cu-64 solution; 3) a custom-made hot-rod resolution phantom containing four groups of hot rods of 0.35, 0.5, 0.75, and 1 mm; and 4) an image quality (IQ) phantom which is the national electrical manufacturers association (NEMA) NU-4 2008 phantom scaled down by 70% [46] with 0.50, 0.80, 1.10, 1.50, and 1.80 mm rod diameters. The resolution phantom and IQ phantom were also filled with Cu-64 solution. The schematic of the resolution phantom is shown as Fig. 9, in which the center-to-center distance of two adjacent rods is 1.6 mm in 1 mm region and other center-to-center distances are twice of the diameters.

For Na-22 point source and Cu-64 resolution phantom, we have rotated the phantom in three angular steps and used the projection acquired at each angular step as a subset for reconstruction. For the line source study, we used 16 angular-steps and the number of events for within the subsets reduces with the subsets acquired at later times due to the decay of Cu-64. The decay is equivalent that we have a constant activity but the sensitivity decreases. Suppose the number of events in each subset is $N_{1},N_{2},\ldots ,N_{\text{sub}}$, the sensitivity $s_{i}$ for each subset is modified as $s_{i}\cdot ([N_{\text{sub}}]∕[N_{1}])$ to account for the decay of source.

## III. Results

### A. Intrinsic Energy Resolution of the CZT Sensor

**Fig. 10.**
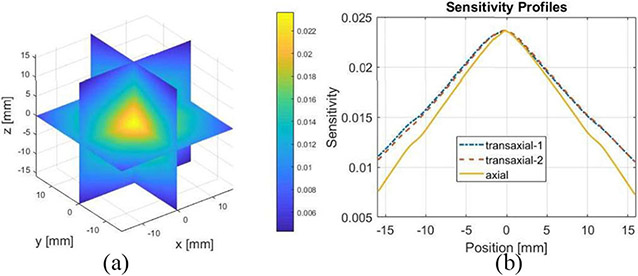
(a) Sensitivity and (b) its profiles of the prototype PET system estimated from modeled system response function.

Using the model detailed in Section II-B, we calculated the sensitivity of the prototype CZT-PET system as shown in Fig. 10. The peak sensitivity of the system is ~ 2.2% at the center of the FOV.

**Fig. 11.**
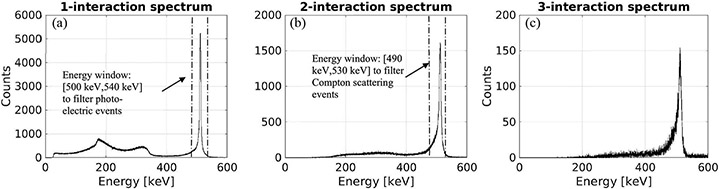
Energy spectra of Cu-64 acquired by CZT detectors. (a) Energy spectrum of gamma-rays detected as one interaction. (b) Spectrum of the total energy of gamma-rays detected as Compton events (two detected interactions). (c) Spectrum of the total energy of gamma-rays detected as Compton events (three detected interactions).

One of the interesting aspects of the large volume 3-D CZT detector is its ability to detect multiple interactions that simultaneously occurred in the detector and precisely determine the position and energy deposition for each individual interaction. The experimentally measured energy spectra from single-site, double-site, and triple-site gamma-ray interactions are shown in Fig. 11(a)-(c). For the double-site and triple-site gamma-ray interactions, we simply summed the energy depositions observed at all interaction sites.

The energy resolution obtained from single-site gamma-ray interactions was 5.4 keV FWHM at 511 keV. With the CZT detector of 2.2 cm × 2.2 cm × 1 cm in size, we observed 80% single-site events, 18% double-site events, 1.5% triple-site events, and the remaining 0.5% of events have more than three interactions.

By looking through the list-mode data collected with the prototype PET setup, we counted the numbers of coincidence events falling into several categories as shown in Table II, which include: 1) single-site 511-keV full-energy interactions on both sides; 2) one of the coincident photon was detected as a Compton scattering event and the total energy deposition falling into the energy window shown in Fig. 11(b), and the other photon was detected as a single-site full-energy event; 3) both coincident photons were detected as Compton events; and 4) all other types of detected coincidence events (e.g., having more than two interactions). This experimental measurement was performed with a custom-made hot-rod resolution phantom with an active volume confined within a cylinder of 1.4-cm diameter and 1-cm length placed at the center of the FOV. The phantom contained Cu-64 solution with an activity of 25 *μ*Ci.

### B. Preliminary PET Imaging Studies With Ideal Coincidence Events With Full-Energy, Single-Site Interactions

**Fig. 12.**
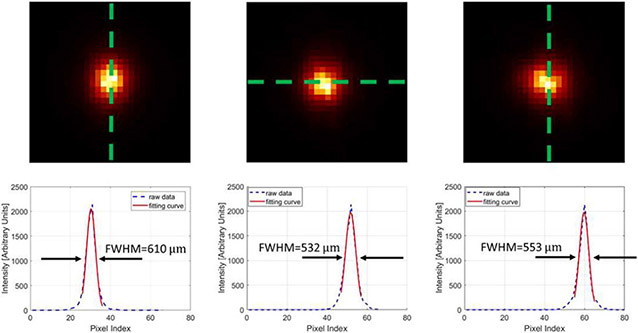
(4th iteration) Three views and line profiles (going through the green dashed line) of the reconstructed Na-22 point source. The object space has 66 × 66 × 80 cubic voxels of 0.1 mm × 0.1 mm × 0.1 mm in size.

**Fig. 13.**
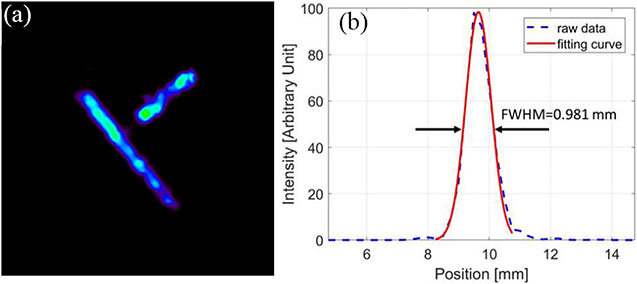
(11th iteration) (a) Reconstructed image of two line sources filled with Cu-64 and (b) its 1-D cross section. The object space has 128 × 128 × 128 cubic voxels of 0.25 mm × 0.25 mm × 0.25 mm in size.

To evaluate the performance of the prototype CZT-PET system, we carried out a series of preliminary imaging studies with a point source, a line source, and a custom-made hot-rod resolution phantom. A Gaussian filter with 0.40-mm FWHM was applied to the reconstructed images of the line sources, the resolution phantom, and the IQ phantom. Fig. 12 shows the image of a Na-22 point source of 0.25-mm diameter reconstructed with 3 subsets of ~18000 events for each, which showed a spatial resolution of approximately 0.5 mm. Fig. 13 shows the reconstructed image of two line sources fabricated with 2 capillary tubes of 0.65-mm inner diameter filled with Cu-64 solution. The FWHM of the 1-D cross section of the reconstructed line-source image is around 0.98 mm. These images are reconstructed with selected coincidence events in which both coincidence gamma-rays interacted in CZT detectors as single-site events, and the energy window of [500 and 540 keV] was used to select these coincidence events.

To further illustrate the spatial resolution attainable with the prototype CZT-PET system, we used a custom-made resolution phantom, as mentioned in Section II-G.

**Fig. 14.**
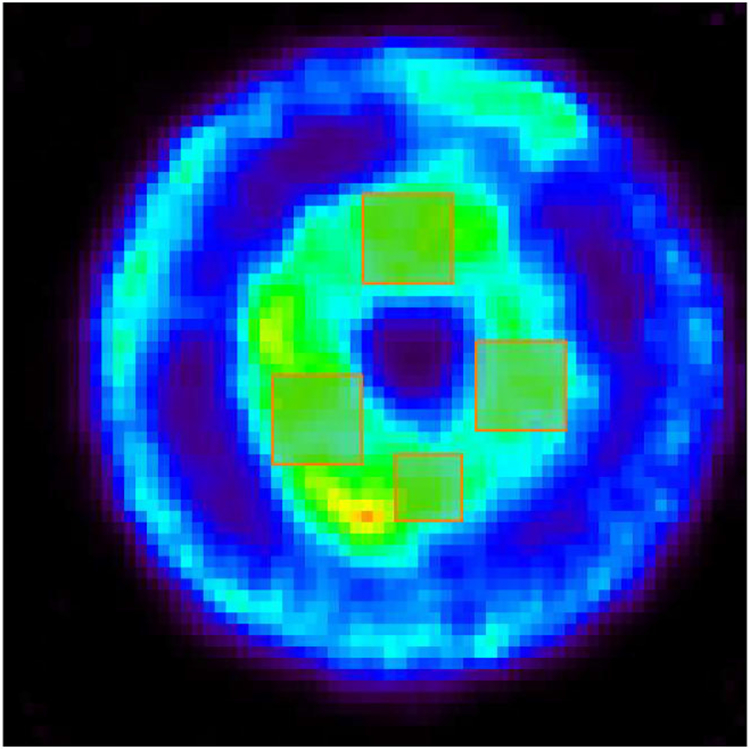
Selected four uniform regions in the hot-rod resolution phantom with a thickness of 1 mm. The total volume 14.25 mm^3^.

With the list-mode coincidence data, we generated several datasets by binning the experimentally derived DOI values with several different DOI bin sizes of 1, 2.5, 5, and 10 mm (no-DOI information used) across the 1-cm detector thickness. Only coincidence events with single-site interactions were used. We used these datasets to demonstrate the impact of DOI resolution in resultant spatial imaging resolution. All phantom images were reconstructed with a list-mode OS-EM algorithm [45]. The number of iterations used for each dataset was chosen to ensure that the resultant images have the same normalized standard deviation (SD) across the preselected regions of interest (ROI), as shown in Fig. 14. The ROIs were chosen in the cylindrical active region on top of the phantom containing continuous Cu-64 solution. Given the possibility that the activity may not be uniform due to the practical sample preparation procedure. We selected four separate ROIs containing relatively uniform activity distribution within their own region. The total volume within the four ROI’s is 14.25 mm^3^. The normalized SD values for the four ROIs were calculated by dividing the SD value by the mean reconstructed activity in the corresponding ROI and the total normalized SD was then calculated by summing the four normalized SD values with mean reconstructed activity weighted.

**Fig. 15.**
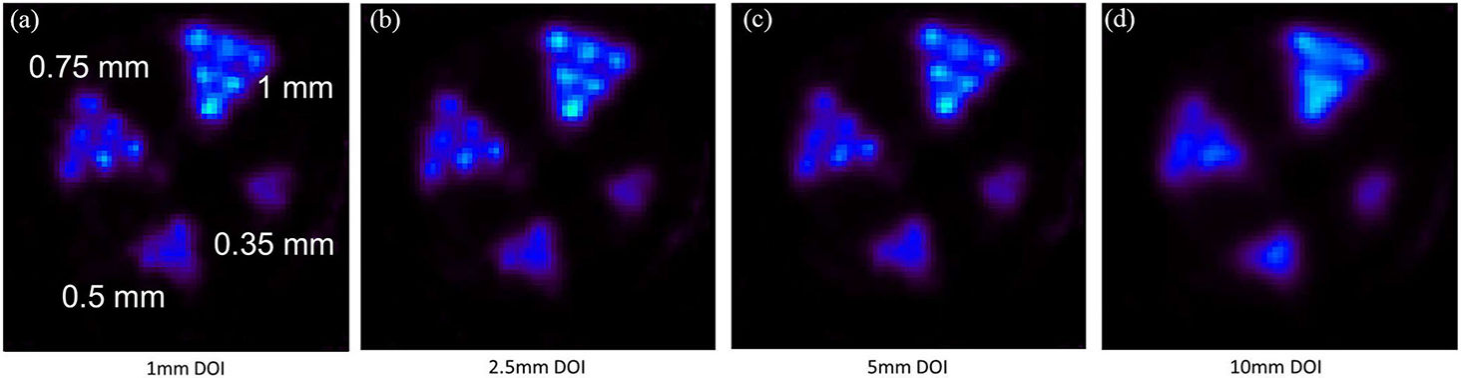
Reconstructed images of hot-rod resolution phantom filled with Cu-64 with DOI resolutions of (a) 1 mm (9th iteration), (b) 2.5 mm (8th iteration), (c) 5 mm (9th iteration), and (d) 10 mm (6th iteration). Only coincidence events with single-site interactions were used in this comparison. The object space has 66 × 66 × 80 cubic voxels of 0.25 mm × 0.25 mm × 0.25 mm in size. These transverse views are of 1-mm thick slices. The normalized SDs of the ROI are all approximately 0.1534.

**Fig. 16.**
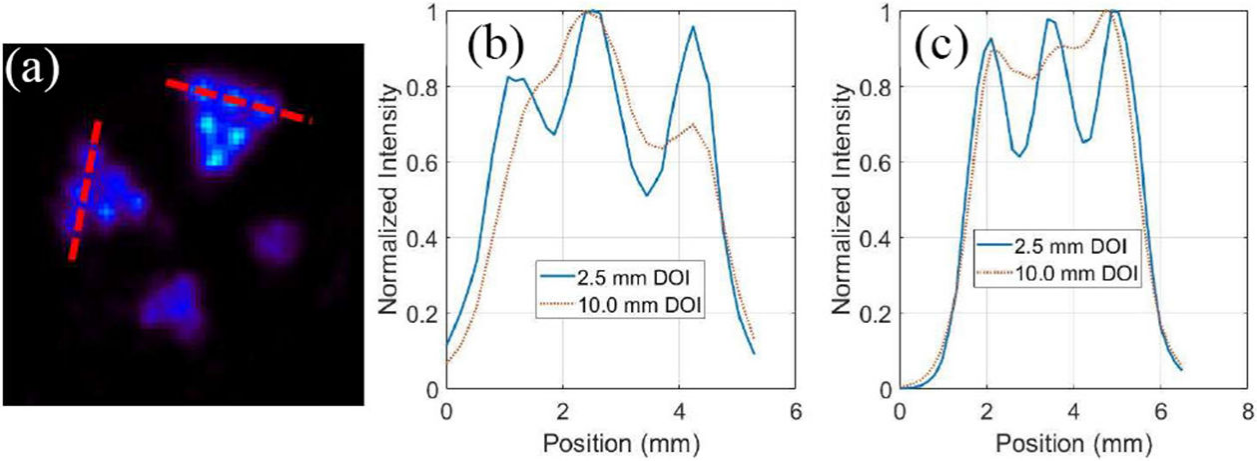
Comparison of line profiles (going through the red dashed lines in (a)) with different DOI resolutions, 2.5 mm (8th iteration), and 10.0 mm 6th iteration), in different phantom regions: (b) 0.75-mm hot-rod region and (c) 1-mm hot-rod region. One-millimeter-thick slices were used.

Fig. 15 compares the PET images of the same resolution phantom reconstructed with the datasets with DOI bin sizes of 1, 2.5, 5, and 10 mm (no DOI information used), respectively. The object space has 66 × 66 × 80 cubic voxels of 0.25 mm × 0.25 mm × 0.25 mm in size. These transverse views are all of 1-mm thick slices. By incorporating the DOI information, we were able to resolve the 0.75-mm hot-rods and the 1-mm hot-rods with 1.6-mm center-to-center distance at different radial offsets. Fig. 16(b) and (c) compares the line profiles going through the two red dashed lines in Fig. 16(a) with different DOI resolutions, which demonstrate the spatial resolution enhancement of using the DOI information.

**Fig. 17.**
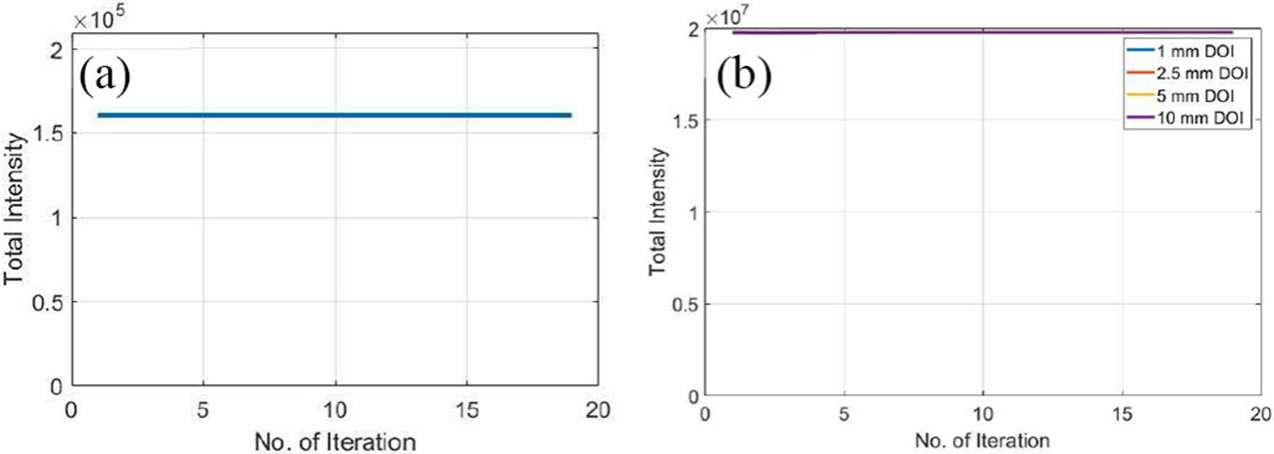
Total intensity of reconstructed images across the process of OS-EM iteration of (a) line sources and (b) hot-rod resolution phantom with different DOI resolutions.

Fig. 17 shows the conservation of total intensity (proportional to the actual counts) of images across the process of OS-EM iteration of line sources and hot-rod resolution phantom with different DOI resolutions, which demonstrates the correct use of the OSEM algorithm.

**Fig. 18.**
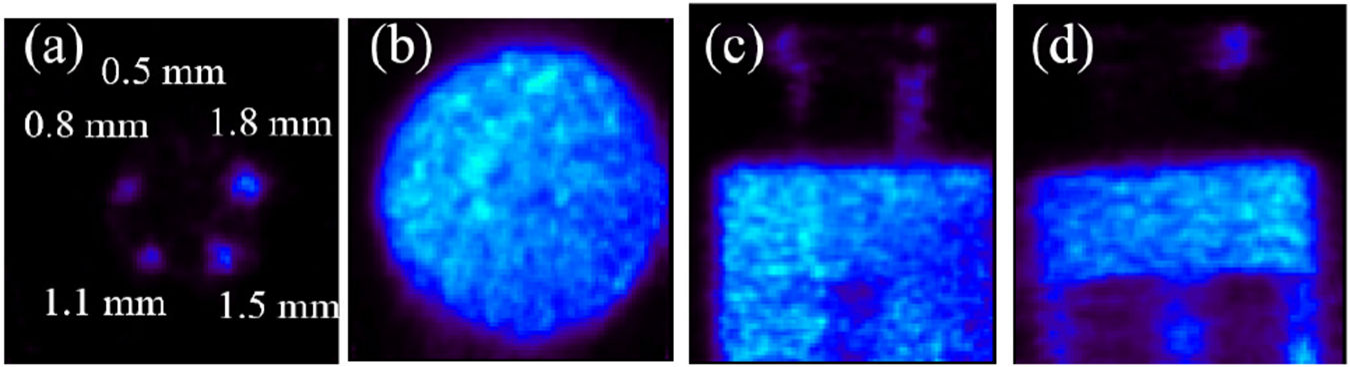
(2nd iteration) Reconstructed image of the IQ phantom filled with Cu-64: (a) transverse view of rod region, (b) transverse view of uniform region, (c) coronal view, and (d) sagittal view. The object space has 80×86×96 cubic voxels of 0.25 mm × 0.25 mm × 0.25 mm in size.

Fig. 18 shows the reconstructed image of the IQ phantom described in Section II-G. The 3-angle acquisition collected approximately 1 million ideal coincidence events with full-energy, single-site interactions, and only 3% of them contributed to the rods region, which makes Fig. 18(a) noisy.

### C. Evaluation of the Accuracy of Compton-Based Random Rejection

**Fig. 19.**
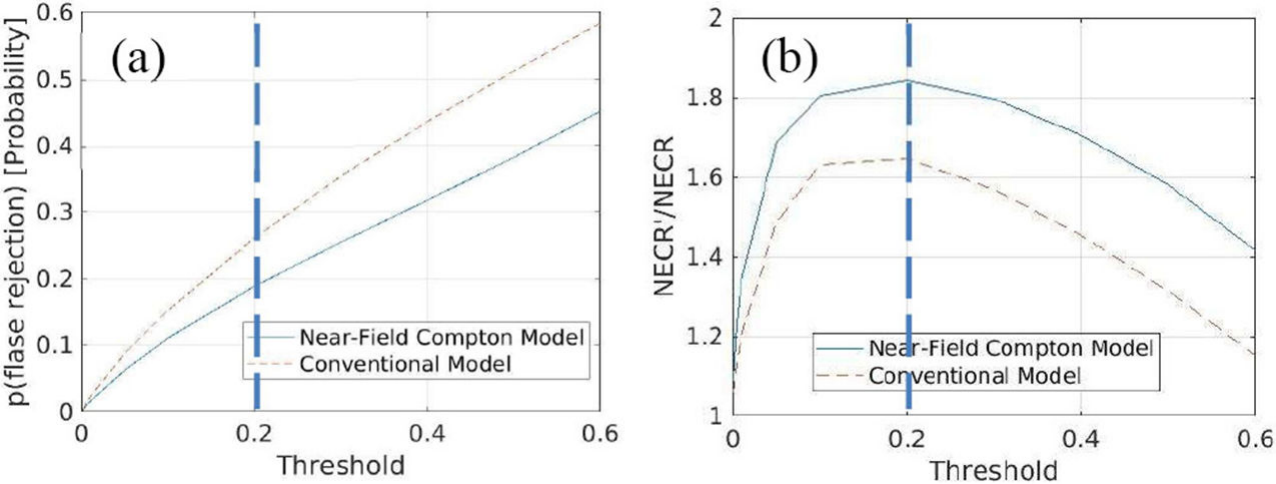
With the near-field Compton model proposed in this study (blue solid lines) and the conventional Compton model without considering ${\hat{d}}_{2}$ (orange dashed line), we evaluated the (a) relationship between the probability of false rejection and the rejection threshold and (b) relationship between NECR′/NECR and the rejection threshold.

As discussed in Section II-D, we evaluated NECR′ of the three subsets of data acquired with the hot-rod resolution phantom and the prototype system to optimize the threshold for Compton-based random and scattered rejection. In (11), *NECR* is an unknown constant, and false rejetion rate is estimated by p (false rejection). Finally, the relationship between NECR′/NECR and the threshold is shown as Fig. 19(b). To get the highest NECR′, 0.2 is set to be the threshold.

With the optimized threshold, we further estimated the true rejection rate using the method mentioned in Section II-D and counted the numbers of coincidence events falling into different categories by processing the three subsets of list-mode data acquired with the hot-rod phantom and the prototype system, which include: 1) single-site 511-keV full-energy interactions on both sides; 2) at least one of the coincident photon was detected as a Compton scattering event and the total energy deposition falling into the energy window shown in Fig. 11(b); 3) accepted events in 2) after Compton-based rejection; 4) events in 2) that their lines of response do not go through the object space (known to be random); and 5) rejected events in 4) after Compton-based rejection. As a result, the near-field Compton model and the rejection method allow us to reject ~67.8% of *chance* coincidence events (true rejection rate), meanwhile ~18% of *true* coincidence events would be rejected incorrectly (probability of false rejection) [Fig. 19(a)]. In total, ~64% of total coincidence events having observed Compton events on at least one of coincident annihilated photons within the current threshold settings. With the conventional Compton model as mentioned in Section II-D and the same dataset, the rejection threshold is still 0.2 and it will reject ~69% of *chance* coincidence events (true rejection rate) but at the same time, it will reject ~26% of *true* coincidence events incorrectly (probability of false rejection) [Fig. 19(a)]. In total, ~66% of total coincidence events having observed Compton events on at least one of coincident annihilated photons. The optimal ratio of NECR′/NECR obtained with the two models are 1.85 and 1.65, respectively [Fig. 19(b)].

### D. Impact of Compton-Based Random Rejection on PET Images

To demonstrate the impact of Compton-based random rejection on the PET IQ, we used the identical experimental coincidence datasets acquired with the prototype CZT-PET setup from the resolution phantom and the IQ phantom, but processed and selected in six different ways.

1. *Dataset 1:* Containing coincidence events with only single-site interactions on both sides.
2. *Dataset 2:* Containing coincidence events with both single-site interactions and double-site interactions within the CZT detectors. The experimentally detected double-site Compton interactions were preprocessed using the Compton kinematics as described in Section II-D to determine the most probable first interaction site and reject random and scattered coincidence.
3. *Dataset 3:* The same collection of coincidence events as in dataset 2 but without Compton-based random rejection. In cases of Compton scattering detected in the CZT sensors, we considered the interaction closer to the cathode of the detector as the first interaction.
4. *Dataset 4:* Inheriting from dataset 2, but randomly discarding the coincidence events with single-site interactions to make the total number of events equal to dataset 1.
5. *Dataset 5:* Inheriting from dataset 4, the coincidence events with double-site interactions in this dataset will be positioned by the energy-weighted centroid method instead of the positioning method in dataset 2.
6. *Dataset 6:* Inheriting from dataset 3, randomly discarding the same number of coincidence events with single-site interactions as dataset 4. In addition, instead of using Compton-based rejection, we randomly discarded the same number of coincidence events with double-site interactions as dataset 2. The double-site interactions will be positioned using energy-weighted centroid method.

Note that for the resolution phantom, the above selection process led to a total of 1.1-million events with both annihilation gamma-rays detected through single-site photoelectric interactions (dataset 1) and 1.2-million coincidence events with at least one of the coincidence gamma-rays detected through a Compton scattering followed by a photoelectric absorption (considered in dataset 3). Among these 1.2-million coincidence events detected through Compton scattering, 0.7-million events were rejected based on Compton kinematics. The total number of events in datasets 1, 4, 5, and 6 are the same, and they all have the same portion of coincidence events with single-site interactions while the extra portion of coincidence events are having: 1) only single-site interactions; 2) Compton events after Compton-based rejection and first interaction prediction; 3) Compton events after Compton-based rejection and energy-weighted centroid positioning; and 4) Compton events after randomly discarding and energy-weighted centroid positioning, respectively.

**Fig. 20.**
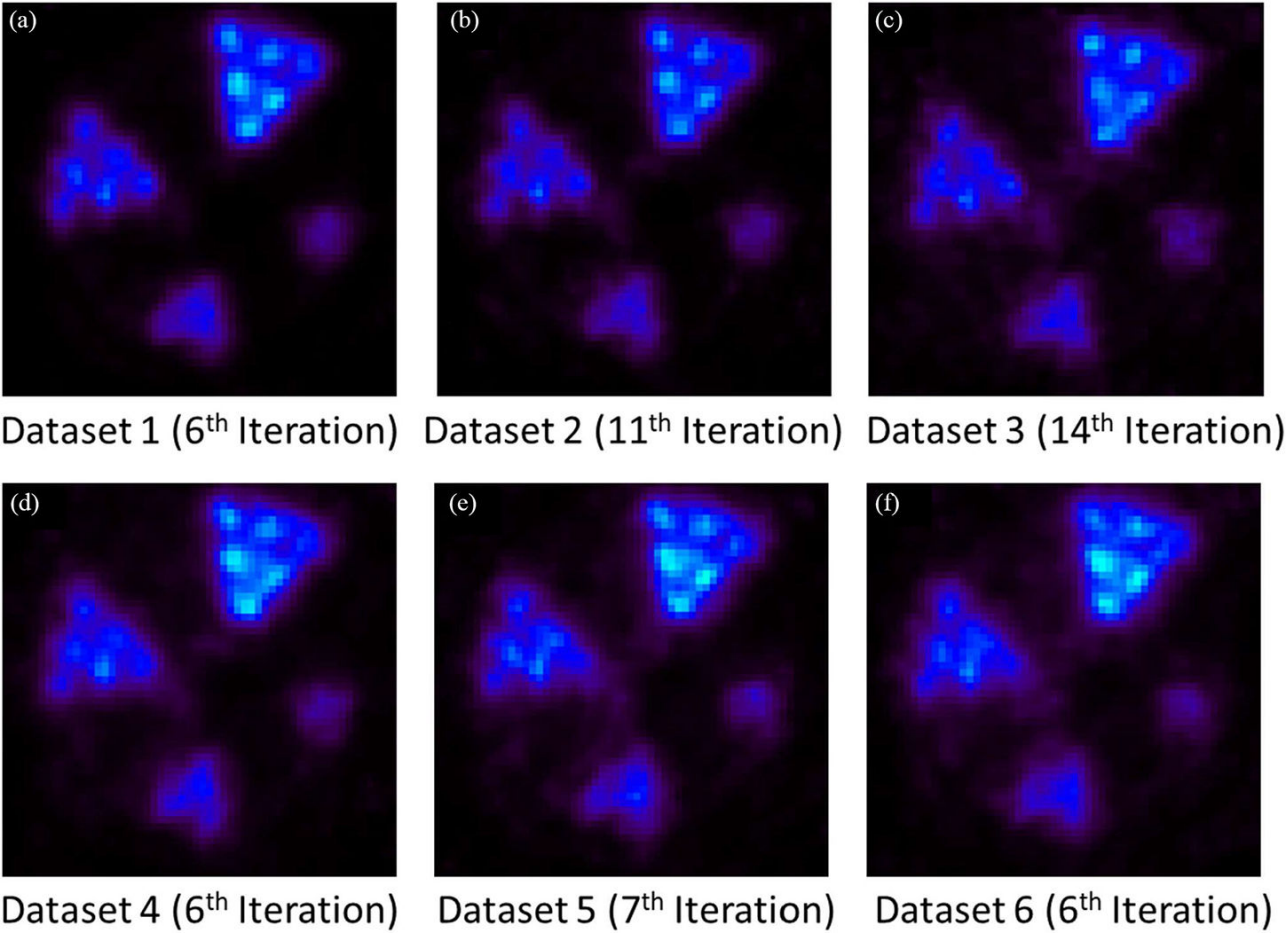
Comparison of reconstructed images of the hot-rod resolution phantom with (a) dataset 1 (6th iteration), (b) dataset 2 (11th iteration), (c) dataset 3 (14th iteration), (d) dataset 4 (6th Iteration), (e) dataset 5 (7th iteration), and (f) dataset 6 (6th iteration) The object space has 66 × 66 × 80 cubic voxels of 0.25 mm × 0.25 mm × 0.25 mm in size. These transverse views are of 1-mm thick slices.

The resolution phantom reconstructed with 0.5-mm DOI resolution and with the above six datasets are shown in Fig. 20(a)-(f). Once again, these images were reconstructed with the OS-EM algorithm but with different iteration numbers to ensure the same SD across the preselected ROIs in Fig. 14.

**Fig. 21.**
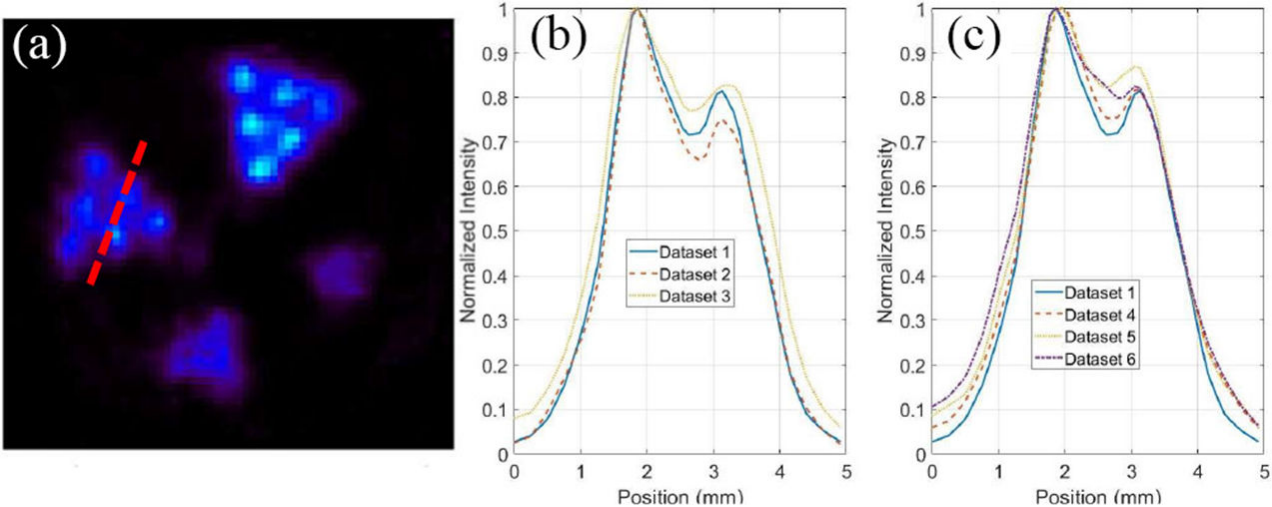
Comparison of line profiles from the 750-*μ*m hot-rods [going through the red dashed line in (a)] of the resolution phantom: (b) reconstructed using datasets 1, 2, and 3 with P/V ratios of 1.13, 1.13, and 1.07; and (c) reconstructed using datasets 1, 4, 5, and 6 with P/V ratios of 1.13, 1.08, 1.05, and 1.02. The profiles are of transverse views. of 1-mm thick slices.

Fig. 21(b) and (c) shows the line profiles going through the red dashed line in Fig. 21(a). With Compton-based rejection [Fig. 20(b)], the IQ is significantly improved compared to the image reconstructed with dataset 3 [without Compton-based rejection, Fig. 20(c)]. We use peak-to-valley (P/V) ratio to quantify the IQ. P/V ratio is defined as the ratio of the height of the lower peak and the height of the valley. In Fig. 21(b), P/V ratios are 1.13, 1.13, and 1.07, respectively, while in Fig. 21(c), P/V ratios are 1.13, 1.08, 1.05, and 1.02, respectively.

**Fig. 22.**
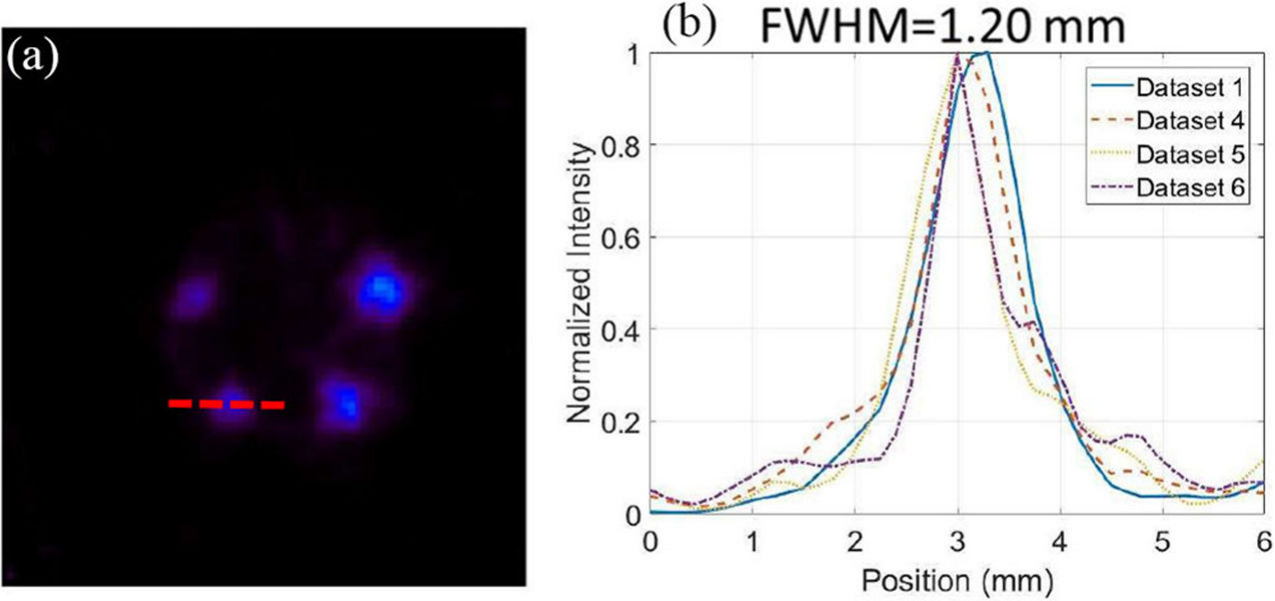
Comparison of line profiles going through 1.10-mm diameter rod (the red dashed line in (a)) of selected images reconstructed by dataset 1 (2nd iteration, dataset 4 (4th iteration), dataset 5 (5th iteration), dataset 6 (6th iteration). With Gaussian fitting, the FWHMs of these peaks are ~1.20 mm. The profiles are of transverse views of 1-mm thick slices.

**Fig. 23.**
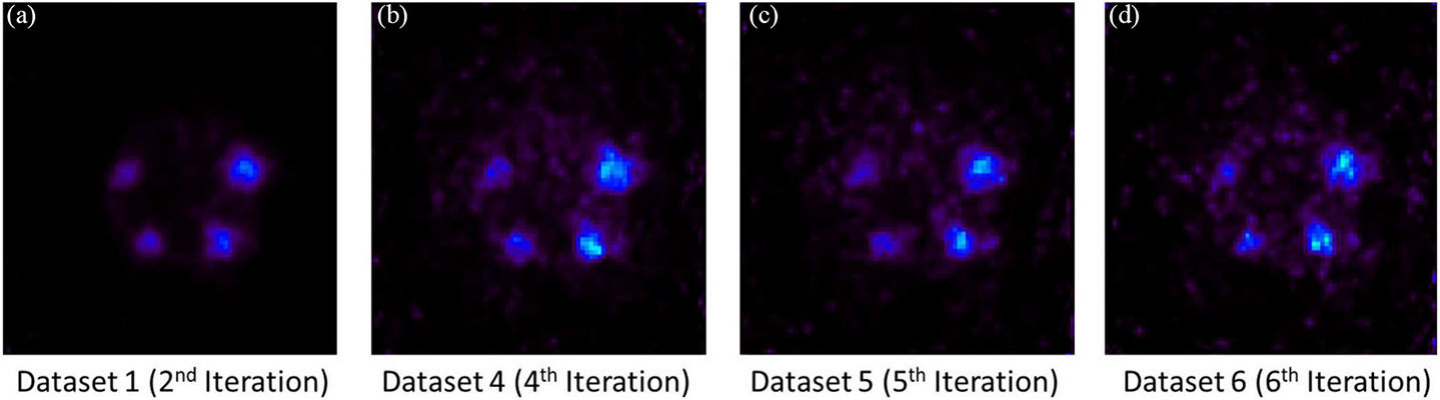
Comparison of reconstructed images of the IQ phantom with (a) dataset 1 (2nd iteration), (b) dataset 4 (4th iteration), (c) dataset 5 (5th iteration), and (d) dataset 6 (6th Iteration). The object space has 80×86 × 96 cubic voxels of 0.25 mm × 0.25 mm × 0.25 mm in size. These transverse views are of 1-mm thick slices.

**Fig. 24.**
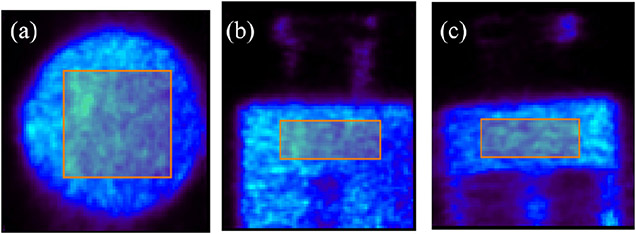
Selected ROI in the uniform region of the IQ phantom with a size of 10.25 mm × 10.25 mm × 4 mm. The total volume is 420.25 mm^3^.

To quantitively compare the influence of Compton-based rejection and first interaction-site prediction on noise property, we used datasets 1, 4, 5, and 6 from the IQ phantom. The number of iterations used to compare was chosen to ensure that the resultant images have similar resolutions at the 1.10-mm diameter hot-rod, as shown in Fig. 22. As shown in Fig. 23, the transverse views of the hot-rods region with different datasets illustrate that with mixing worse-defined Compton events, the images become noisier, especially for Fig. 23(d) in which Compton events were not applied Compton-based rejection. We selected the ROI in the uniform region of the phantom as shown in Fig. 24, with a total volume of 420.25 mm^3^. The computed normalized SDs of this selected ROI for datasets 1, 4, 5, and 6 are 0.136, 0.182, 0.214, and 0.238, respectively.

## IV. Discussion

We studied the IQ offered by the prototype PET system, in particular, the spatial resolution of the system with the DOI-enabled CZT imaging sensors. The current prototype offers a peak sensitivity of 2.2%, but the effective FOV is confined by the 4-cm width of the CZT detector panel to a cylindrical volume of a few cm in diameter.

With the prototype CZT-PET system geometry shown in Fig. 1 and the small phantom used in the imaging study, the DOI information is less important, since the parallax error is relatively small, considering that most gamma rays would reach the detectors at a relatively small angle of incidence. Nevertheless, incorporating the DOI information in the reconstruction did lead to an improved spatial resolution over the imaging results without DOI information (Figs. 15 and 16). The images of point source, line source, the resolution phantom, and the IQ phantom is shown in Figs. 12, 13, 15, and 18 have demonstrated an imaging resolution of ~ 0.75 mm.

One of the keys to effective random rejection based on Compton data is an accurate detector Compton response. In particular, the response function would capture both the idealized Compton interaction physics and the imperfection in realistic CZT detectors, such as the realistic energy and spatial resolution and the finite dimension of the accurate sensor. In this work, we presented a Monte-Carlo-based numerical integration technique to derive the Compton response function that not only considers the Compton kinematics (including Doppler broadening), but also the finite detector spatial and energy resolutions, as well as the short distances between the first and second interaction sites practically observed in the CZT detector of 1-cm thickness. By comparison, the previous efforts only considered highly idealized Compton response functions that simply ignore the effect of Doppler broadening, finite detector resolutions, and the short distances between the interaction sites in detectors. The comprehensive system model that we implemented in this work led to a much-improved accuracy in predicting the near-field Compton response of the CZT detectors.

Among the coincidence events detected with the prototype PET setup, 48% were detected with single interactions on both sides, and 52% with at least one of the coincident gamma-ray detected through Compton scattering followed by a photoelectric absorption. As we discussed in Section III-C, we were able to reject 64% of the coincidence events having at least one of the coincident gamma-ray detected through Compton scattering followed by a photoelectric absorption under the rejection threshold of 0.2. The threshold is optimized by maximizing NECR which is a PET performance metric and has a strong correlation with the rejection threshold. We have also quantified the accuracy of our Compton-based rejection method by estimating the probability of false rejection of 0.18 and the true rejection rate of ~0.678. Compared to the model proposed in this study, using the conventional Compton model in rejection will reject more chance coincidence events (true rejection rate of ~0.69) and much more true coincidence events (probability of false rejection rate of ~0.26), which makes the NECR 11% lower than using our near-field Compton model.

In Fig. 20(b), we have demonstrated that the 3-D CZT detectors, with their excellent spatial and energy resolutions, could allow us to use Compton kinematics to select the first interaction site and to partially reject random coincidences, which increases the sensitivity by ~35% without spatial resolution degradation compared to Fig. 20(a). In addition, there is a noticeable improvement in imaging resolution [shown in Fig. 20(b)] over the image obtained without applying the Compton-based data corrections [shown in Fig. 20(c)]. By controlling the total number of coincidence events, we compared the influence of coincidence events having: 1) only single-site interactions; 2) Compton events after Compton-based rejection and first interaction prediction; 3) Compton events after Compton-based rejection and energy-weighted centroid positioning; and 4) Compton events after randomly discarding and energy-weighted centroid positioning, on the resultant spatial resolutions and noise properties with the hot-rod resolution and the IQ phantom. With 1), the reconstructed image shows the best spatial resolution while from 2) to 4), the artifacts and spatial resolution are getting worse and worse, which demonstrates the benefits of the first interaction site prediction, Compton-based rejection, and near-field Compton model proposed in this study. Furthermore, the comparison of the normalized SDs of the selected regions in Fig. 24 quantitively demonstrates the improvement of noise properties and the superiority of our methods.

Note that the random rate, or noise, of coincidence events having single-site interactions only (dataset 1) is much lower than that of dataset 3 in which we included coincidence events having two-interaction events on at least one side, as shown in Fig. 20(a) and (c). This is because in dataset 1, only the interactions having energy deposition within the energy window of [500 and 540 keV] (Fig. 11) is used, in which case only two random photons deposited their full energy through photoelectric effect could lead to random coincidence event. However, the sum of energy deposition is used to filter two-interaction events, in which case, other than at least one of two random photons deposited their full energy through Compton scattering followed by a photoelectric absorption, three or four random photons could also lead to random coincidence by combining their partial energy deposition. Therefore, the chance of random coincidence having two-interaction events is higher than that having one-interaction events only.

## V. Conclusion

In this study, we have experimentally evaluated a prototype PET system based on large-volume 3-D CZT detectors that offer an excellent intrinsic spatial resolution of around 0.5 mm, an excellent energy resolution of around 5.4 keV at 511 keV, and the ability to detect multiple gamma-ray interactions simultaneously occurred in the detector. This unique hardware system allowed us to detect Compton interactions inside the CZT detectors and apply Compton kinematics to identify the first Compton interaction site and reject random and scattered coincidences.

We have developed a numerical integration technique to model the near-field Compton response of the CZT detectors that incorporates the influence of the distance between the first and second interactions. We have also used point sources, line sources, a custom-designed resolution phantom, and a commercial IQ phantom to demonstrate an imaging resolution of around 0.75 mm in PET images. With the experimental data acquired from the hot-rod resolution phantom, we have selected the threshold of Compton-based rejection by optimizing NECR and made an estimation of rejection accuracy in terms of the probability of false rejection and true rejection rate. Through this study, we have demonstrated that the use of the Compton kinematics allowed us to reject a substantial portion of random and scattered coincidences and significantly improve the PET IQ compared to results obtained without Compton-based data corrections.

Note that the current CZT-PET prototype has a relatively poor timing resolution due to the specific timing signal readout approach. This issue could be potentially alleviated by using a cathode-signal-based technique that allows one to achieve a significantly improved timing resolution [39], [40]. By comparison, scintillation detectors deliver an excellent timing resolution, count-rate capability, and intrinsically lower Compton fraction than CZT detectors. To effectively utilize the Compton information to reject random coincidence in CZT detectors, the sensors would need to be equipped with advanced electronics and specific detector design, which are both under development.

The excellent spatial resolution and energy resolution of the CZT sensors would also allow for Compton imaging across a wide energy range. This possibility will be explored in our future studies.

## Figures and Tables

**TABLE I:** Characterization of Detectors in Recent Experimental Research on CZT/CdTe-Based PET System

Research	Method	FWHM Energy Res. at 511 keV	FWHM Spatial Res.	FWHM DOI Res.	Detecting Multiple Interaction Capability
Prototype CZT-based PET scanner [6][7]	Simulation	2%	1 mm	0.5 mm	Not report
Brain PET scanner using CdTe detectors [8]	Experiment	4%	> 4 mm	> 1 mm	Yes
CdTe strip detector for PET [11]	Simulation	3%	0.5 mm	2 mm	Not report
Dedicated head and neck PET based on CZT detectors [12][13]	Simulation	4%	Not report	Not	Not
PET system based on cross-strip CZT detectors [14][15][16][17][18]	Experiment Simulation	7.8%	0.44 mm	Not	Yes
Ultra-high resolution CdTe-based PET [19] [20]	Experiment	5%	0.35 mm	0.25 mm	No
VP-PET [21] [22] [23] [24]	Experiment and simulation	10%	0.35 mm	Not report	Not report
This Work	Experiment	1%	0.5 mm	0.5 mm	Yes

**TABLE II:** Numbers of Detected Coincidence Events With Different Types

Number of coincidence events (Proportion)	Single interactions observed on both sides	Observed Compton on one side	Observed Compton on both sides	All other coincidence events
Out of energy window	2,611,308 (87.8%)	1,186,913 (78.5%)	204,292 (77.1%)	72,394 (82.6%)
Within the energy window	362,290 (12.2%)	325,279 (21.5%)	60,663 (22.9%)	15,246 (17.4%)
Total	2,973,598 (100%)	1,512,192 (100%)	264,955 (100%)	87,640 (100%)

**TABLE III:** Numbers of Detected Coincidence Events in Three Subsets

Number of coincidence events	Single-site interactions on both sides	Compton events on at least one side	Accepted Compton events on at least one side after Compton rejection	Random and having Compton events on at least one side	Compton-Rejected Random and having Compton events on at least one side	True rejection rate
Subset 1	362,290	381,010	137,446	202,597	137,428	0.678
Subset 2	368,748	387,051	140,546	195,405	132,754	0.677
Subset 2	363,536	380,385	137,518	187,673	127,681	0.679
